# Oxazinomycin arrests RNA polymerase at the polythymidine sequences

**DOI:** 10.1093/nar/gkz782

**Published:** 2019-09-09

**Authors:** Ranjit K Prajapati, Petja Rosenqvist, Kaisa Palmu, Janne J Mäkinen, Anssi M Malinen, Pasi Virta, Mikko Metsä-Ketelä, Georgiy A Belogurov

**Affiliations:** 1 Department of Biochemistry, University of Turku, FIN-20014 Turku, Finland; 2 Department of Chemistry, University of Turku, FIN-20014 Turku, Finland

## Abstract

Oxazinomycin is a C-nucleoside antibiotic that is produced by *Streptomyces hygroscopicus* and closely resembles uridine. Here, we show that the oxazinomycin triphosphate is a good substrate for bacterial and eukaryotic RNA polymerases (RNAPs) and that a single incorporated oxazinomycin is rapidly extended by the next nucleotide. However, the incorporation of several successive oxazinomycins or a single oxazinomycin in a certain sequence context arrested a fraction of the transcribing RNAP. The addition of Gre RNA cleavage factors eliminated the transcriptional arrest at a single oxazinomycin and shortened the nascent RNAs arrested at the polythymidine sequences suggesting that the transcriptional arrest was caused by backtracking of RNAP along the DNA template. We further demonstrate that the ubiquitous C-nucleoside pseudouridine is also a good substrate for RNA polymerases in a triphosphorylated form but does not inhibit transcription of the polythymidine sequences. Our results collectively suggest that oxazinomycin functions as a Trojan horse substrate and its inhibitory effect is attributable to the oxygen atom in the position corresponding to carbon five of the uracil ring.

## INTRODUCTION

**Figure 1.**
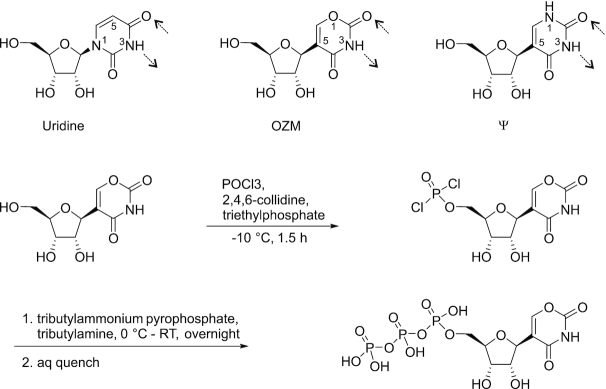
The synthesis of oxazinomycin triphosphate. The chemical structures of uridine, oxazinomycin (OZM) and pseudouridine (Ψ) are shown for comparison. The potential Watson–Crick bonds with adenine are indicated by dashed arrows.

C-nucleosides are a group of compounds that comprise the non-canonical nucleobases capable of Watson–Crick base pairing attached to the unaltered ribose moieties via a C–C bond (as opposed to an N–C bond in canonical nucleosides). Compounds with O- and N-glycosidic bonds are widely present in metabolic pathways, but are enzymatically labile thereby facilitating the rapid recycling of metabolites and the base excision repair of the DNA (1–3). Many biologically active natural products also contain O- and N-glycosidic bonds, but these tend to make the molecules susceptible to hydrolysis in the acidic environment of the stomach and by the action of glycosidases in the small intestine (4). In contrast, C-glycosylation provides an enzymatically resistant and chemically stable bond (5). The best-known C-nucleoside is pseudouridine (Ψ, Figure 1), which supports the functioning of structural RNAs, such as tRNAs and rRNAs, in all cellular lifeforms. It has been shown to stabilize higher order RNA structures through enhanced base stacking (6). Ψ is introduced into RNAs post-transcriptionally and has been implicated in altering the translation of some mRNAs in eukaryotes (7,8). C-glycosidic bonds are also found in several bioactive secondary metabolites (9,10). In particular, *Streptomyces* soil bacteria have been reported to produce C-nucleoside compounds such as pseudouridomycin, formycin, showdomycin and malayamycin (11–14).

Oxazinomycin (OZM; 5-β-d-ribofuranosyl-l,3-oxazin-2,4-dione), also known as minimycin, is a C-ribonucleoside antibiotic first isolated from *Streptomyces hygroscopicus* (*Streptomyces sp. 80432*) (15–17). Tymiak *et al.* also reported its production from *Pseudomonas paucimobilis* (18), and the full chemical synthesis of OZM has been described (19). OZM is active against both gram positive and gram negative bacteria, and has antitumor activity against transplantable tumors (15,16). OZM closely resembles uridine but features an oxygen atom in the position corresponding to carbon five of uridine in addition to the C-glycosidic bond (Figure 1). Considering the known promiscuity of the nucleoside and nucleotide kinases (20), OZM may be converted into the OZM triphosphate upon entry into the target cell, compete with UTP as an RNAP substrate and inhibit transcription or post-transcriptional processes upon its incorporation into RNA. To test this hypothesis, we studied the effects of the OZM triphosphate on transcription by the classic representatives of DNA-dependent RNA polymerases: multisubunit RNAPs from *Escherichia coli* and *Saccharomyces cerevisiae* (RNA polymerase II) and a single subunit RNAP from human mitochondria.

All RNAPs responsible for transcribing cellular genomes are multisubunit enzymes that share homologous catalytic cores. The bacterial RNAP, a five-subunit complex, ααββ’ω, is the simplest model system used for studying the fundamental mechanistic properties of all multisubunit RNAPs. Eukaryotic cellular RNAPs consist of 12–17 subunits and contain homologs of all five subunits present in the less complex bacterial RNAPs (21). In contrast, the single-subunit RNAPs that transcribe mitochondrial genomes are homologous to DNA polymerases and the bactreriophage T7 RNA polymerase (22). The multi- and single-subunit RNAPs display many mechanistic similarities as a result of convergent evolution. Thus, all transcribing RNAPs maintain an 8–10 base pair RNA:DNA hybrid during transcription elongation, undergo closure and opening of the active site by mobile protein domains during catalysis, efficiently discriminate between the four nucleobases, and are specific for ribonucleoside-triphosphates (NTPs) (reviewed in (23,24)).

RNAP translocates along the DNA as a Brownian ratchet (25–29). The translocation equilibrium is intrinsically biased forward at many sequence positions (30–32). However, the multisubunit RNAPs often move backward along the template DNA (backtrack) (33,34) in situations where forward movement is inhibited by an unfavorable template DNA sequence (35), a DNA bound protein (36–38), a lesion (39,40)) or following a misincorporation event (41). Upon backtracking, one or several nucleotides at the 3′ end of the nascent RNA unpair from the template DNA. A single unpaired nucleotide can be accommodated within the active site (42–45), whereas backtracking by many nucleotides extrudes the unpaired nucleotides into the secondary channel of RNAP (46,47), a route for NTP substrate entry into the active site. The recovery of the backtracked RNAP into a catalytically competent state is facilitated by the endonucleolytic cleavage of the extruded nascent RNA. The cleavage reaction is catalyzed by the active site of RNAP (48) with the assistance of the dissociable factors GreA and B in bacteria (49,50), TFS in archaea (51,52) and eukaryotes (53,54), or the mobile domains of specialized subunits in the case of the eukaryotic Pol I and Pol III (55). In contrast, backtracking by the single subunit RNAPs has not been extensively documented. The experimental evidence is limited to a study by Zamft *et al.* who reported the stimulation of mitochondrial transcription by secondary structures in the nascent RNA (56). These observations are consistent with backtracking by the mitochondrial RNAP but are arguably insufficient to prove that backtracking does take place. In addition, a computational study by Da *et al.* inferred that the mitochondrial RNAP may be more prone to backtracking than the homologous bacteriophage T7 enzyme (57). Finally, no cleavage assisting factors analogous to bacterial Gre factors have been identified for the mitochondrial RNAP.

Bacterial RNAP is a validated target for antibacterial drugs (reviewed in (58–60)). Several classes of antimicrobials function by selectively inhibiting the bacterial RNAP. These compounds can be divided into at least nine functional classes exemplified by rifamycin (61–64), streptolydigin (65,66), microcin J25 (67,68), myxopyronin (69,70), salinamide (71), GE23077 (72), CBR703 (73–75), fidaxomicin (76–78) and pseudouridimycin (79). Among the antimicrobials listed above only pseudouridimycin is a bona fide nucleoside analog inhibitor, which consists of an N-hydroxylated Gly–Gln dipeptide conjugated to 5′-amino-pseudouridine. However, pseudouridimycin does not undergo phosphorylation and does not incorporate into RNA: the dipeptide tail mediates selective binding of the antibiotic in the active site of bacterial RNAPs by functionally mimicking the triphosphate moiety (79). Here, we show that triphosphorylated OZM is a good substrate for multi- and single-subunit RNAPs but incorporation of several successive OZMs by the multisubunit RNAPs leads to backtracking along the template DNA and transcriptional arrest. Our results suggest that OZM functions as a transcriptional inhibitor if present at similar or higher concentration than uridine in the cell, but do not rule out the possibility that OZM may interfere with post-transcriptional processes such as RNA folding and translation when the intracellular OZM/U is low.

## MATERIALS AND METHODS

### Reagents and oligonucleotides

HPLC-purified DNA oligonucleotides were purchased from Eurofins Genomics GmbH (Ebersberg, Germany). The PAGE purified ATTO680 labeled RNA primer was purchased from IBA Biotech (Göttingen, Germany). NTPs and Ψ triphosphate (ΨTP) were from Jena Bioscience (Jena, Germany). DNA oligonucleotides and RNA primers are presented in Supplementary Table S1. All other reagents used were molecular biology grade.

### Oxazinomycin production and isolation

*Streptomyces hygroscopicus* subsp. *hygroscopicus* JCM 4712 was cultivated in the medium containing 4.0 % glucose, 2.0% soybean meal (Cereform Ltd, Northampton, England), 1.0% wheat embryo (Ravintoraisio Oy, Raisio, Finland), 0.4% brewer’s yeast (Lucullus Backen & Geniesssen GmbH & Co KG, Germany), 0.2 % NaCl, 0.2% KCl and 0.2% CaCO_3_ (16). Precultivation was performed in 50 ml of media in 250-ml Erlenmeyer flasks with shaking for 3 days at 30°C. For production, 39 flasks were inoculated with 0.5 % of the preculture and cultivated as described above. After 2 days of cultivation, a 5 ml sample of the culture medium was centrifuged to remove bacterial cells and compounds from the supernatant were absorbed to 0.1 g of charcoal overnight at 4°C. Compounds were extracted from the charcoal with 1 ml of acetone, 0.5 ml sample of the extract was evaporated to dryness, dissolved in 200 μl of H_2_O and analyzed with LC-MS using Agilent Technologies 1260 Infinity Binary LC and Agilent 6100 Series Quadrupole LC/MS Systems equipped with Phenomenex 150 × 4.6 Synergi™ 4 μm Fusion-RP 80 Å analytical column. The flow rate was 0.5 ml/min, the absorbance detector wavelength was set to 210 nm, 0.1 % formic acid in H_2_O and MeCN were used as eluents. Following the detection of a compound corresponding to the mass of OZM by LC-MS (see above), the supernatant (1.7 l) was collected by centrifugation and pH was adjusted to 2.0 with HCl. Activated charcoal (20 gl^−1^) was added to the supernatant and the mixture was stirred overnight at 4°C. The charcoal was collected by filtration and the adsorbed compounds were washed out of the charcoal cake with acetone/H_2_O (1:1) (1 L). The combined extracts were concentrated to a small volume and the mixture was fractioned by silica gel chromatography, eluting first with acetone/toluene (2:8) and then with acetone/toluene (8:2). The fractions containing OZM were combined and evaporated to dryness under reduced pressure. The residue was dissolved in a small volume of water and OZM was finally isolated by RP-silica column chromatography (length 125 mm, diameter 25 mm) eluting with a gradient of 3–6 % MeCN/H_2_O. The fractions containing OZM were evaporated to dryness and further dried in a vacuum desiccator to yield 184 mg of OZM as a white solid. ^1^H NMR (500 MHz, D_2_O): *δ* 7.73 (s, 1H, C=CH), 4.60 (d, *J* = 4.9 Hz, 1H, H-1′), 4.19 (pseudo-t, *J* = 4.9 Hz, 1H, H-2′), 4.05 (pseudo-t, *J* = 5.7 Hz, 1H, H-3′), 3.92 (m, 1H, H-4′), 3.76 (dd, *J* = 3.1 and 12.5 Hz, 1H, H-5′), 3.63 (dd, *J* = 5.0 and 12.5 Hz, 1H, H-5″). ^13^C NMR (125 MHz, D_2_O): *δ* 163.0 (C-2), 154.6 (C-6), 149.7 (C-4), 114.2 (C-1), 83.1 (C-4′), 78.0 (C-1′), 73.5 (C-2′), 70.5 (C-3′), 61.3 (C-5′); HRMS (ESI) *m/z*: [M-H]^-^ calculated for C_9_H_10_NO_7_^-^ 244.0457; found 244.0465. The chemical structure validation data are presented in Supplementary Figure S1.

### Synthesis of OZM triphosphate

OZM (36 mg, 0.15 mmol) was dissolved in dry triethyl phosphate (TEP) (0.95 ml) under nitrogen atmosphere and the solution was cooled down to −10°C on an ice-salt bath. Freshly distilled phosphoryl chloride (22 μl, 0.24 mmol) was added dropwise and then dry 2,4,6-trimethylpyridine (20 μl, 0.15 mmol) in one portion. The reaction was allowed to proceed for 1.5 h at −10°C, then a dry solution of tributylammonium pyrophosphate (265 mg, 0.29 mmol) in MeCN (1.5 ml) and dry tributylamine (70 μl, 29 mmol) were added to the cooled reaction mixture, and the stirring was continued for 2 h at 0°C, and then for 18 h at room temperature. Triethylammonium acetate solution (2.5 ml, 0.05 M, pH 7.0) and chloroform (2.5 ml) were added to the reaction mixture and stirring was continued for 30 min. The aqueous layer was separated and washed two times with chloroform (3 ml). NaI (80 mg) and acetone (35 ml) were added to the aqueous solution to precipitate the phosphorylated compounds and the mixture was vortexed for 15 min and cooled down to 0°C. The precipitate was collected by centrifuging the mixture and separating the supernatant. The OZM 5′-triphosphate was purified by RP-HPLC (Phenomemex 250 × 10 Kinetex™ 5 μm C18 100 Å column, flow rate 3 ml min^−1^, eluted with a gradient from 0.025 M triethylammonium acetate in H_2_O to 0.025 M triethylammonium acetate in acetonitrile/H_2_O, 1:1, over 25 min). The collected fractions were lyophilized to yield a triethylammonium salt of the OZM 5′ triphosphate. ^1^H NMR (500 MHz, D_2_O): *δ* 7.82 (d, 1H, *J* = 1.2 Hz, H6), 4.73 (overlaps with H_2_O, 1H, H-1′), 4.20 (m, 1H, H-2′), 4.23 (m, 1H, H-3′), 4.07 (m, 1H, H-4′), 4.16 (m, 1H, H-5′), 4.09 (m, 1H, H-5″); ^13^C NMR (126 MHz, D_2_O): *δ* 163.6 (C-2), 154.5 (C-6), 150.2 (C-4), 114.7 (C-1), 81.3 (C-4′), 77.6 (C-1′), 74.0 (C-2′), 69.9 (C-3′), 64.8 (C-5′); ^31^P NMR (202 MHz, D_2_O): *δ* – 10.74 (d, *J* = 19.9 Hz, P-*γ*), – 11.32 (d, *J* = 19.9 Hz, P-*α*), – 23.36 (pseudo-t. *J* = 19.9 Hz, P-*β*); HRMS (ESI) *m/z*: [M-H]^-^ calcd for C_9_H_13_NO_16_P_3_^-^ 483.9453; found 483.9451. The chemical structure validation data are presented in Supplementary Figures S2 and S3.

### Proteins

*Escherichia coli* (*Eco*) RNAP was expressed in T7 Express *lysY/I^q^* cells from New England Biolabs (Ipswich, MA, USA) bearing the pVS10 plasmid (or pIA528 and pIA839 plasmids in case of β’N458S RNAP) and was purified by Ni-, heparin and Q-sepharose chromatography as described previously (80), dialyzed against the Storage Buffer (50% glycerol, 20 mM Tris-HCl pH 7.9, 150 mM NaCl, 0.1 mM EDTA, 0.1 mM DTT) and stored at −20°C. *Saccharomyces cerevisiae* RNA polymerase II (*Sce* RNAPII) was purified from the *S. cerevisiae* strain SHy808 (kindly provided by the laboratory of Mikhail Kashlev, NIH, National Cancer Institute, Frederick, MD, USA) largely as described previously (81,82).

The human mitochondrial RNAP (*Hsa* MT RNAP) lacking 213 N-terminal amino acids (the mitochondrial localization signal and an unstructured regulatory domain (83)) was expressed in *E. coli* T7 Express *lysY/I^q^* cells bearing the pGB163 plasmid. The cells were grown in 1 L LB medium supplemented with 50 μg/ml kanamycin at 37°C until OD 0.6, the culture was transferred to 25°C, protein expression was induced for 5 h by the addition of 0.8 mM IPTG. Cells were harvested by centrifugation at 6000 × *g*, 4°C for 10 min, resuspended in Lysis Buffer (50 mM Tris-HCl pH 6.9, 500 mM NaCl, 5% glycerol) supplemented with 1 mM β-ME, a tablet of EDTA-free protease inhibitors (Roche Applied Science, Mannheim, Germany), 1 mg/ml lysozyme, incubated on ice for 30 min and disrupted by sonication. The lysate was cleared by centrifugation at 18 000 × *g*, 4°C for 30 min. The supernatant was supplemented with 10 mM imidazole and loaded onto a Ni-sepharose (GE Healthcare, Chicago, IL, USA) column pre-equilibrated with Lysis Buffer. Protein was eluted using a step gradient (20, 50, 250 mM) of imidazole in Lysis Buffer. The 250 mM imidazole fraction containing RNAP was further purified using Heparin and Resource-S columns in Buffer A (50 mM Tris-HCl pH 6.9, 5% glycerol, 1 mM β-mercaptoethanol, 0.1 mM EDTA) and Buffer B (Buffer A supplemented with 1.5 M NaCl). *Hsa* MT RNAP eluted at ≥50% and ≥30% Buffer B from Heparin and Resource-S columns, respectively. The fractions containing the purified protein were concentrated using Amicon Ultra-4 centrifugal filters (Merck Milipore, Burlington, MA, USA), dialyzed overnight in Storage Buffer (10 mM Tris-HCl pH 7.5, 50% glycerol, 100 mM NaCl, 0.1 mM EDTA, 0.1 mM DTT) and stored at −80°C.

*Escherichia coli* GreA and GreB were expressed in *E. coli* T7 Express *lysY/I^q^* cells bearing the pIA578 (GreA) or the pIA577 (GreB) plasmid and purified by Ni-sepharose followed by gel filtration as described in Perederina *et al.* (84). The obtained protein was dialyzed against the Storage Buffer (50% glycerol, 20 mM Tris-HCl pH 7.9, 1 M NaCl, 0.1 mM EDTA, 0.1 mM DTT) and stored at −20°C. The plasmids used for protein expression are listed in Supplementary Table S2.

### TEC assembly

*Eco* and *Hsa* MT RNAP TECs (1 μM) were assembled by a procedure developed by Komissarova *et al.* (85). An RNA primer was annealed to the template DNA, incubated with 1.5 μM RNAP for 10 min in TB10 buffer (40 mM HEPES-KOH pH 7.5, 80 mM KCl, 10 mM MgCl_2_, 5% glycerol, 0.1 mM EDTA, and 0.1 mM DTT) and with 2 μM of the non-template DNA for 20 min at 25°C. *Sce* RNAPII TECs were assembled as described above except that the incubation time after the addition of the non-template DNA was 10 min.

### *In vitro* transcription reactions, single nucleotide addition assay

The transcription reactions were initiated by the addition of 10 μl of NTP in TB10 buffer to 10 μl of the assembled TEC in TB10 buffer. The final concentrations of the TEC and NTP were 0.1 and 20 μM, respectively. The reactions were incubated for 2 min at 25°C and quenched with 30 μl of Gel Loading Buffer (94% formamide, 20 mM Li_4_-EDTA and 0.2% Orange G). RNAs were separated on 16% or 25% denaturing polyacrylamide gels and visualized with an Odyssey Infrared Imager (Li-Cor Biosciences, Lincoln, NE, USA); band intensities were quantified using the ImageJ software (86).

### *In vitro* transcription reactions, processive transcript elongation

The transcription reactions were initiated by the addition of 10 μl of NTP mixtures in TB10 buffer to 10 μl of the assembled TEC in TB10 buffer. Three different mixtures of NTPs contained ATP, CTP, GTP and UTP (U-chase) or OZM triphosphate (OZM-chase) or ΨTP (Ψ-chase). The final concentrations of the TEC and NTPs were 0.5 and 100 μM each, respectively. The reactions were incubated for 5 min at 25°C and quenched with 40 μl of Gel Loading Buffer. RNAs were separated on 16% denaturing polyacrylamide gels, visualized and quantified as described above.

### GreA facilitated RNA cleavage

TEC were prepared by incubating the assembled TEC (1 μM) with 10 μM UTP and GTP or OZM triphosphate and GTP in TB10 for 3 min at 25°C and passed through Zeba™ Spin desalting columns 40K MWCO (Pierce Biotechnology, Rockford, USA) pre-equilibrated with TB0 buffer (40 mM HEPES-KOH pH 7.5, 80 mM KCl, 5% glycerol, 0.1 mM EDTA and 0.1 mM DTT). RNA cleavage was initiated by mixing 10 μl of the pre-extended TEC with 90 μl of GreA in TB10 at 25°C. The final concentrations of the TEC, GreA and Mg^2+^ were 0.1, 2 and 9 mM, respectively. Aliquots (10 μl) were withdrawn at the indicated time points and quenched with 30 μl of Gel Loading Buffer. RNAs were separated on 16% denaturing polyacrylamide gels, visualized and quantified as described above.

### Time-resolved measurements of nucleotide addition

Time-resolved measurements of nucleotide addition were performed in an RQF 3 quench-flow instrument (KinTek Corporation, Austin, TX, USA). The reaction was initiated by the rapid mixing of 14 μl of 0.2 μM TEC with 14 μl of 400 μM NTP. Both TEC and NTP solutions were prepared in TB10 buffer. The reaction was allowed to proceed for 0.004–60 s at 25°C and quenched by the addition of 86 μl of 0.45 M EDTA or 0.5 M HCl. HCl quenched reactions were immediately neutralized by adding 171 μl of neutralizing-loading buffer (94% formamide, 290 mM Tris base, 13 mM Li4-EDTA, 0.2% Orange G). RNAs were separated on 16% denaturing polyacrylamide gels, visualized and quantified as described above.

### Equilibrium fluorescence measurements

Equilibrium levels of fluorescence were determined by recording the emission spectra of 6-methyl-isoxanthopterin (6-MI) (excitation at 340 nm) with an LS-55 spectrofluorometer (Perkin Elmer, Waltham, MA, USA) at 25°C. The fluorescence at the peak emission wavelength (420 nm) was used for the data analysis and representation. Preassembled TECs (1 μM) were diluted into 200 μl of TB10 buffer with or without NTPs and guanosine-5′-[(α,β)-methyleno]triphosphate (GMPCPP). The final concentrations of the TEC, NTPs and GMPCPP were 0.1, 10 and 500 μM, respectively. Following a 2-min incubation at 25°C, 5 μl aliquots were withdrawn, quenched with 20 μl of the Gel Loading Buffer and subsequently analyzed in a denaturing PAGE to determine the RNA extension efficiency. The rest of the sample was transferred into a 16.160-F/Q/10 quartz cuvette (Starna Scientific Ltd, Essex, UK) and the emission spectra were recorded.

### Time-resolved fluorescence measurements

Measurements were performed in an Applied Photophysics (Leatherhead, UK) SX.18MV stopped-flow instrument at 25°C. The 6-MI fluorophore was excited at 340 nm and the emitted light was collected through a 400-nm longpass filter. The reaction was initiated by mixing 60 μl of 0.2 μM TEC with 60 μl of 2.5–500 μM of substrate NTP (both in TB10). At least three individual traces were averaged for each concentration of NTP.

### Data analyses

Time-resolved uridine and Ψ incorporation data (HCl and EDTA quenched reactions) and the translocation time-traces were simultaneously fitted to a four-step model using the numerical integration capabilities of the KinTek Explorer software (87) (KinTek Corporation, Austin, TX, USA) as described previously (88,89). The model postulated that the initial TEC16 slowly and reversibly interconverts between the inactive and active states, the active state reversibly binds the NTP substrate, undergoes an irreversible transition to TEC17 upon incorporation of the nucleotide into RNA, followed by the irreversible translocation. The first reversible isomerization step was needed to account for a small (∼10%) fraction of the slow TEC that incorporated nucleotides more than 10-fold slower than the fast fraction. This step was omitted from the reaction schematics presented in the results section where only a three-step scheme is depicted for simplicity. Given that the translocation was modeled as an irreversible transition, the inferred translocation rate corresponds to the sum of the forward and backward translocation rates (88) as further discussed in the results section. The EDTA quenched reactions were modeled using the pulse-chase routine of the KinTek explorer software during the global analysis of the data. Thus, EDTA quenches the nucleotide addition reactions by chelating Mg^2+^, thereby inactivating the free NTP substrate, but allows a fraction of the RNAP-bound NTP to incorporate into RNA after the addition of the quencher (90). The latter fraction corresponds to the ratio of the nucleotide addition rate to the sum of the nucleotide addition rate and the rate of NTP dissociation from the RNAP active site. As a result, the inclusion of the EDTA quench time courses into the analysis allowed for the estimation of the rate of UTP and ΨTP dissociation from the RNAP active site.

The time courses of guanosine incorporation were fitted to a sum of exponential (modeled fast phase) and stretched exponential (modeled slow phase) functions. The time courses of the GreA facilitated cleavage of the nascent RNA were fitted to the stretched exponential function. The median reaction times were calculated as in Turtola & Belogurov (91). We used the stretched exponential function in the analyses because it is the simplest analytical function that robustly describes the slow processes in transcription that are, in most cases, poorly described by the single and double exponential functions (45,91).

## RESULTS

### OZM production, purification and the synthesis of OZM triphosphate

*Streptomyces hygroscopicus* subsp. *hygroscopicus* JCM 4712 was cultivated in the medium described by Kusakabe *et al.* (16). After 3 days of cultivation, a compound corresponding to the mass of OZM was detected with LC-MS, extracted from the culture media with activated charcoal and purified by chromatographic methods. The chemical structure of the isolated compound was evaluated by NMR and HRMS spectroscopy and the obtained spectroscopic data (see ‘Materials and Methods’ section) matched the parameters reported for OZM (17–19). The purified OZM was converted into the 5′-triphosphate via Yoshikawa’s phosphorylation (92) using phosphoryl chloride followed by treatment with the nucleophilic pyrophosphate salt and, finally, aqueous hydrolysis (Figure 1). RP-HPLC purification using triethylammonium acetate buffered eluents yielded the triethylammonium salt of the OZM 5′-triphosphate.

### OZM efficiently incorporates into RNA in place of uridine

**Figure 2.**
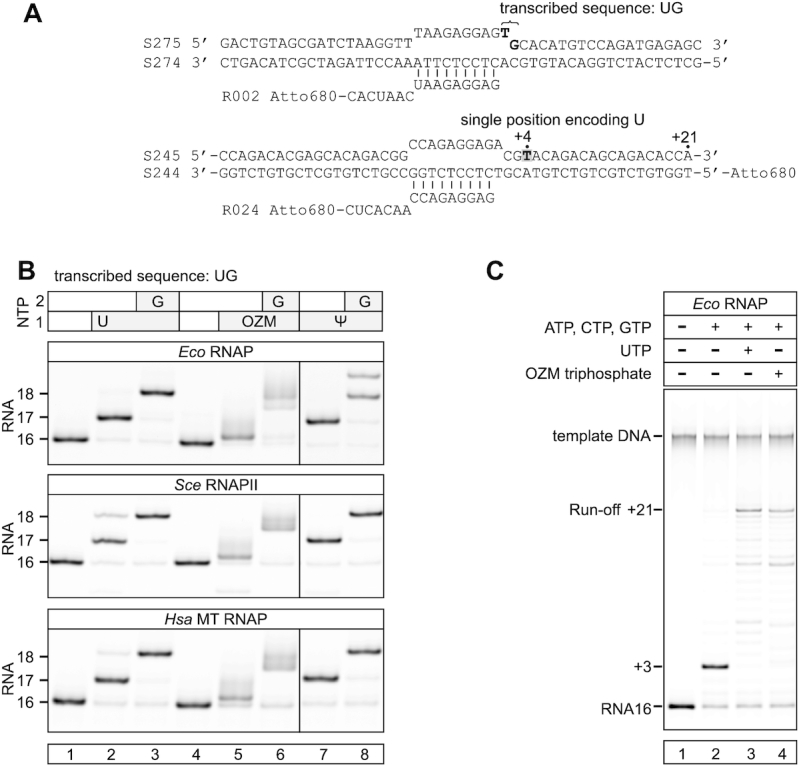
*Eco* RNAP, *Sce* RNAPII and *Hsa* MT RNAP efficiently incorporate OZM in place of uridine. (**A**) The nucleic acid scaffolds employed in the experiments in (B) and (C). (**B**) Incorporation of OZM in place of uridine: the initial TECs (lanes 1 and 4; top schematics in (A)) were supplemented with UTP and GTP (lanes 2 and 3), OZM triphosphate and GTP (lanes 5 and 6), ΨTP and GTP (lanes 7 and 8). NTPs were added at 20 μM and the reactions were incubated for 2 min at 25°C. RNAs were resolved on 25% urea PAGE. Fractional misincorporations (additional bands) are evident in several lanes. (**C**) OZM triphosphate allows efficient transcription of the sequence position encoding uridine: the initial TEC (lane 1, bottom schematics in (A)) was supplemented with ATP, CTP and GTP (lane 2) and additionally with UTP (lane 3) or OZM triphosphate (lane 4). NTPs were added at 100 μM and the reactions were incubated for 15 s at 25°C. The limited read through the position encoding uridine in the absence of UTP and OZM triphosphate (lane 2) was likely due to the misincorporation of CMP in place of UMP. Each assay was performed in triplicate. Pixel counts were linearly scaled to span the full 8-bit grayscale range within each gel panel.

We first tested if OZM can be incorporated into RNA in place of uridine by the multisubunit RNAP from *E. coli* (*Eco*), RNA polymerase II from *S. cerevisiae* (*Sce* RNAPII) and the single subunit RNAP from human mitochondria (*Hsa* MT RNAP). Transcription elongation complexes (TEC) were assembled on synthetic nucleic acid scaffolds and contained a fully complementary transcription bubble flanked by 20-nucleotide long DNA duplexes upstream and downstream (Figure 2A). The annealing region of a 16-nucleotide RNA primer was 9 nucleotides. The RNA primer was 5′ labeled with the infrared fluorophore ATTO680 to monitor RNA extension by denaturing PAGE. We first employed a TEC that incorporates uridine followed by guanosine. The addition of UTP and UTP+GTP to the TEC resulted in significant changes in the mobility of the RNA in the PAGE gel as expected (Figure 2B, lanes 1–3). In contrast, the employment of the OZM triphosphate in place of UTP resulted in a very small change in the electrophoretic mobility of the RNA (Figure 2B, lanes 4 and 5). However, the mobility of the RNA changed as if it was extended by two nucleotides upon the addition of the OZM triphosphate and GTP (Figure 2B, lane 6). These observations indicated that OZM efficiently incorporated into RNA in place of uridine but caused only a very small change in its electrophoretic mobility.

To test if the small effect on electrophoretic mobility is a general property of C-nucleosides, we performed the experiments with Ψ triphosphate (ΨTP) in place of UTP. Incorporation of Ψ into RNA altered the electrophoretic mobility to a similar extent as did the incorporation of uridine (Figure 2B, lanes 7–8) suggesting that the small change in the electrophoretic mobility upon OZM incorporation was not due to the C-glycosidic bond, but was likely attributable to the oxygen atom in the position corresponding to carbon five of the uracil ring (Figure 1). Qualitatively similar results were obtained with *Eco* RNAP, *Sce* RNAPII and *Hsa* MT RNAP (Figure 2B).

We next tested if OZM can be incorporated in place of cytidine, adenine and guanine. *Eco* RNAP and *Hsa* MT RNAP but not *Sce* RNAPII incorporated OZM in place of cytidine (Supplementary Figure S4A, top row). However, the incorporation of OZM in place of cytidine was inefficient, particularly at low OZM concentrations (Supplementary Figure S4B). None of the RNAPs in our test incorporated OZM in place of adenine or guanine (Supplementary Figure S4A, middle and bottom rows). These results suggested that OZM is exclusively a uridine analogue.

To test if OZM can substitute for uridine during processive transcript elongation, we transcribed a short DNA template using mixtures of NTP substrates containing either UTP or OZM triphosphate. In a control experiment, RNAP failed to transcribe a position encoding uridine and stopped at position +3 when supplemented with ATP, CTP and GTP (Figure 2C, lane 2). At the same time, RNAP transcribed to the end of the template when supplemented with the mixture of all four natural NTPs (Figure 2C, lane 3) or ATP, CTP, GTP and OZM triphosphate (Figure 2C, lane 4). These results strongly suggest that OZM incorporates into RNA in place of uridine and permits further extension of the RNA chain following the binding of the next incoming NTP.

### Incorporation of OZM results in incomplete translocation of the RNAP along the DNA

**Figure 3.**
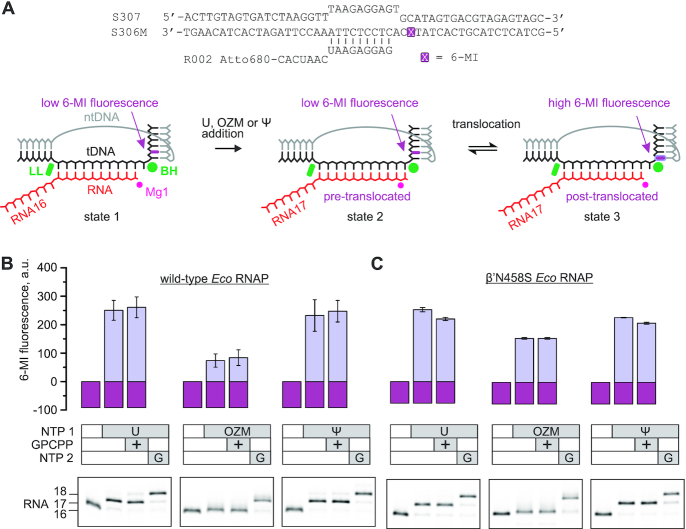
The effect of OZM on translocation by *Eco* RNAP. (**A**) The nucleic acid scaffold employed in the translocation assay. The guanine analog 6-MI was initially positioned in the downstream DNA two nucleotides downstream of the active site. The 6-MI fluorescence was quenched by the neighboring base pairs in the initial TEC (state 1) and the pre-translocated TEC that formed following the nucleotide incorporation (state 2) but increases when the 6-MI relocates to the edge of the downstream DNA upon translocation (state 3). The Bridge Helix (BH) and the Lid loop (LL) are two structural elements of β’ subunit that flank the RNA:DNA hybrid in the multisubunit RNAPs. (**B**) The fluorescence intensities observed upon incorporation of the uridine or OZM or Ψ by TECs assembled with the wild-type *Eco* RNAP. Gel panels reveal the length of the RNA at each step. The incorporation of OZM resulted in a very small change in the mobility of the RNA but was verified by further extension of the TEC with the guanosine. (**C**) The experiments were performed as in (B), but the TECs were assembled using β’N458S *Eco* RNAP.

We next studied the translocation of the *Eco* RNAP along the DNA following OZM incorporation. To assess the effect of OZM incorporation on the translocation bias, we utilized a TEC with the fluorescent base analog 6-methyl-isoxanthopterin (6-MI) incorporated into the template DNA downstream of the active site (Figure 3A). 6-MI closely resembles guanine, forms three Watson–Crick bonds with cytosine and causes minimal disruptions to the DNA duplex (93,94). The TEC was assembled on a synthetic nucleic acid scaffold and contained the fully complementary transcription bubble flanked by 20-nucleotide DNA duplexes upstream and downstream. The annealing region of a 16-nucleotide RNA primer was initially 9 nucleotides, permitting the TEC extended by one nucleotide to adopt the post- and pre-translocated states, but disfavoring backtracking. The RNA primer was 5′ labeled with the infrared fluorophore ATTO680 to monitor the RNA extension by the denaturing PAGE.

In the initial TEC, the fluorescence of 6-MI was strongly quenched by the neighboring bases in the downstream DNA (Figure 3A, state 1). Upon incorporation of a uridine and the forward translocation, 6-MI migrated to the edge of the downstream DNA (Figure 3A, state 3) with a concomitant 3-fold increase in the fluorescence (Figure 3B, left set of bars). The addition of GMPCPP, the nonhydrolyzable analog of the next substrate NTP, did not increase the fluorescence of the uridine extended TEC (TEC-U) suggesting that the forward translocation was complete, i.e. the fraction of the pre-translocated state (Figure 3A, state 2) was smaller than the margins of the error of the fluorescent measurements (∼10%). However, our data did not rule out the possibility that the GMPCPP was unable to measurably alter the translocation bias of the TEC-U (see below).

We next performed the fluorescence experiments with the OZM triphosphate in place of UTP. The amplitude of the increase in the fluorescence upon OZM incorporation (Figure 3B, middle set of bars) was 3-fold lower than it was upon the incorporation of the uridine. However, the addition of GMPCPP did not change the fluorescence of the OZM extended TEC (TEC-OZM) suggesting that the forward translocation was completed, but that the fluorescence of the fully translocated TEC-OZM was lower than that of the TEC-U. Similar effects were observed upon OZM and uridine incorporation by the TEC with the 6-MI fluorophore at the upstream edge of the RNA:DNA hybrid (Supplementary Figure S5). However, we were uncertain if the GMPCPP could shift the translocation bias of the TEC-OZM and used an alternative approach to evaluate the completeness of translocation. To this end, we repeated the translocation experiment using β’N458S *Eco* RNAP where the pre-translocated state was destabilized by a substitution of the β’N458 residue that binds the 3′ and 2′ OH groups of the RNA 3′ end in the pre-translocated state (95). The amplitude of the increase in fluorescence upon extension of the β’N458S TEC with OZM was approximately 70% of the amplitude measured for the uridine extended β’N458S TEC (Figure 3C). This difference was considerably smaller than the 3-fold effect observed with the wild-type TECs (Figure 3B) suggesting that the translocation of the TEC-OZM was incomplete (a measurable fraction of the TEC-OZM was not post-translocated), but 0.5 mM GMPCPP could not shift the translocation bias in our system.

Finally, we evaluated if the incomplete translocation upon OZM incorporation was characteristic for C-nucleosides in general by performing the translocation measurements with ΨTP in place of UTP. The amplitudes of the increase in fluorescence upon the incorporation of Ψ and U by the wild-type RNAP were indistinguishable within the margin of the experimental uncertainty suggesting that the incomplete translocation upon OZM incorporation was not due to the C-glycosidic bond, but was likely attributable to the oxygen atom in the position corresponding to carbon five of the uracil ring (Figure 1).

### OZM triphosphate is largely indistinguishable from UTP as a substrate for the *Eco* RNAP

**Figure 4.**
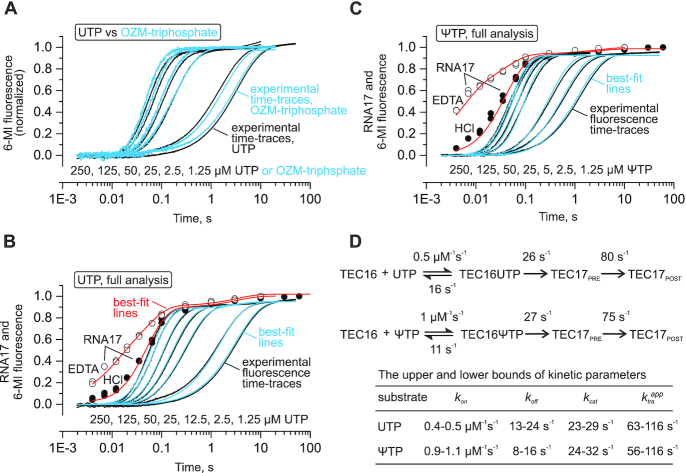
A comparison of UTP, OZM triphosphate and ΨTP as *Eco* RNAP substrates. The experiments were performed using the nucleic acid scaffold presented in Figure 3. (**A**) Time-resolved measurements of the increase in the 6-MI fluorescence upon incorporation of the uridine (back) and OZM (blue). (**B** and **C**) Complete quantitative dissection of the uridine (B) and the Ψ (C) incorporation kinetics by the combined analysis of the 6-MI fluorescence and the RNA extension data. Individual time-points show RNA extension at 200 μM UTP or ΨTP in the quench flow experiments with EDTA (open circles) or HCl (closed circles) as quenchers. Black time-traces show the increase in 6-MI fluorescence in a stopped flow experiments at decreasing UTP or ΨTP concentrations indicated below the curves. The experiments were performed in duplicate, fluorescence curves are averages of three to seven time traces. Best-fit lines for quench flow (red) and stopped flow (blue) experiments were simulated using the scheme and parameters presented in (D). (**D**) The kinetic schemes used for global analyses of the uridine and Ψ incorporation data. The best-fit values of the reaction rates are indicated for each transition. The upper and the lower bounds of the reaction rates calculated at a 10% increase in Chi^2^ over the minimal value using the FitSpace routine of the KinTek Explorer software are presented in the table below the schemes. The translocation was modeled as an irreversible process and the inferred rate constants (*k*_tra_^app^) are the sums of the forward and backward translocation rates.

We next investigated how OZM triphosphate compares with UTP in terms of the affinity for RNAP and the maximal incorporation rate. Due to a very small change in the electrophoretic mobility upon the incorporation of the OZM, we could not use the rapid chemical quench flow method to determine the maximal OZM incorporation rate and the apparent affinity of OZM triphosphate for the RNAP. Instead, we compared the uridine and OZM incorporation using the fluorescence translocation assay (Figure 3A) performed in a stopped flow instrument (30,89). The concentration series of the uridine and OZM incorporation largely superimposed suggesting that the OZM triphosphate was indistinguishable from the UTP as a substrate for the *Eco* RNAP (Figure 4A). To investigate whether the differences in the translocation bias described in Figure 3 could complicate the interpretation of the fluorescence experiments and challenge the conclusions above, we performed a comprehensive analysis of the uridine incorporation kinetics.

We performed the time-resolved measurements of the uridine incorporation in a quench flow setup using HCl and EDTA as quenchers. The combined analysis of the quench flow and the stopped flow data allowed us to fully resolve the uridine incorporation kinetics (Figure 4B and D, see ‘Materials and Methods’ section for details). These analyses suggested that the translocation step caused only a small delay in the completion of the uridine addition cycle at saturating concentrations and could be completely disregarded at low substrate concentrations.

We further inferred that the delay between the nucleotide addition curves (that could not be measured) and the normalized translocation curves (Figure 4A) should be even smaller in the case of OZM incorporation. First, we reasoned that the differences in the translocation bias in Figure 3 were likely due to the higher backward translocation rate in the TEC-OZM because the forward translocation rate is usually determined by the intrinsic stability of the closed active site rather than the nature of the incorporated nucleotide (30,88). Second, we inferred that the same forward and the higher backward translocation rates following the OZM incorporation should make the delay between the OZM addition and the normalized fluorescence time trace smaller. Albeit somewhat unintuitively, yet following the rules of the formal kinetics, the delay is inversely proportional to the sum of the forward and backward translocation rates (88). Considering the inferences described above, we concluded that the differences in the translocation bias following the incorporation of OZM and uridine (Figure 3) were unlikely to affect the conclusion about the equality of the OZM triphosphate and UTP as substrates for the *Eco* RNAP.

Finally, we took the opportunity to completely resolve the kinetics of Ψ incorporation (Figure 4C and D). The analyses suggested that ΨTP approximately equals UTP in terms of the incorporation and translocation rates but binds to the active site 2-fold faster than UTP. The comparative analysis of Ψ and uridine incorporation kinetics highlighted our capabilities to resolve relatively small differences between the similar substrates thereby reinforcing our inference of the equality of the OZM triphosphate and UTP as substrates for *Eco* RNAP.

### Incorporation of a single OZM does not slow down the incorporation of the next nucleotide

**Figure 5.**
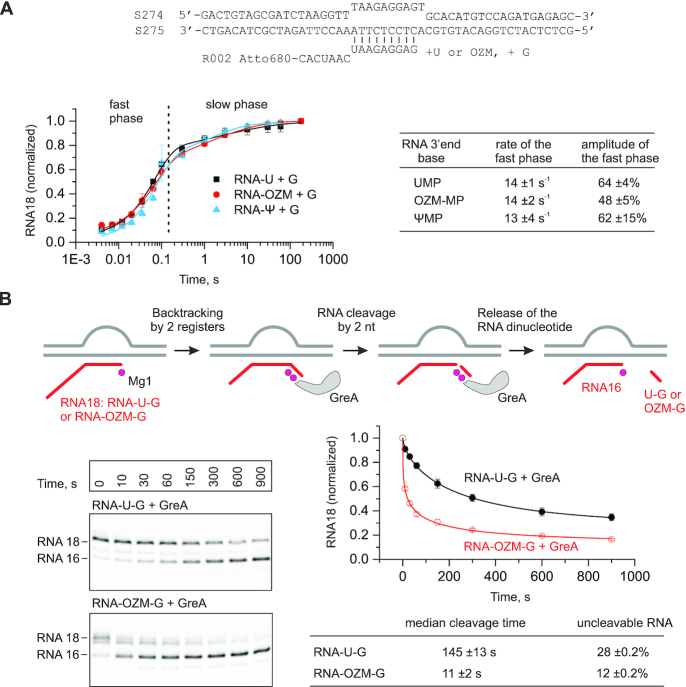
The effect of the OZM at the RNA 3′ end on the kinetics of the incorporation of the next nucleotide (**A**) and the GreA mediated RNA cleavage of the resulting TEC (**B**). (A) TECs were pre-extended by the addition of 10 μM UTP (black), OZM triphosphate (red) or ΨTP (blue) and supplemented with 200 μM GTP in quench flow experiments. Error bars are ranges of duplicate measurements and the solid lines are the best-fits to a sum of exponential (corresponds to the fast phase) and stretched exponential (corresponds to the slow phase) functions. The rates and the amplitudes of the fast phase inferred from the data in the graph are presented in the table on the right. The TEC schematic is presented above the graph. (B) TECs were assembled as in (A) and pre-extended with uridine and guanosine or OZM and guanosine by the addition of 10 μM of the corresponding NTPs, gel filtrated and supplemented with 2 μM of GreA. Error bars are ranges of duplicate measurements and the solid lines are the best-fits to a stretched exponential function. The median cleavage times and the fractions of RNA resistant to GreA-mediated cleavage inferred from the data are presented in the table below the graph.

We next investigated how the incorporation of OZM into RNA affects the rate of the incorporation of the next nucleotide. The assembled TECs were pre-extended with uridine (TEC-U) or OZM (TEC-OZM) or Ψ (TEC-Ψ) and challenged with 200 μM GTP in a quench flow instrument. The resulting guanosine incorporation time courses largely superimposed (Figure 5A) suggesting that the TEC-U, TEC-OZM and TEC-Ψ incorporated guanosine with similar rates. Fitting the time courses to the biexponential function confirmed this prediction (Figure 5A, table on the right) and suggested a rate of ∼14 s^−1^ for guanine incorporation by the fast fraction (50–60%) of the TEC-U, TEC-OZM and TEC-Ψ. The slow fraction of the TECs that incorporated guanosine with the rate of ∼1 s^−1^ (40–50%) likely represented a population of paused TECs that is usually present in TEC preparations, though typically does not exceed 15–20% (30,88). The rate of guanosine incorporation by the slow fraction was too slow to associate this fraction with the equilibrium between the pre- and post-translocated states. Fractional backtracking was also unlikely because the RNA–DNA complementarity was artificially limited to ten base pairs in the TEC-U, TEC-OZM and TEC-Ψ. Overall, the guanosine incorporation experiments indicated that the altered translocation bias in the TEC-OZM (Figure 3) did not affect the observed guanosine incorporation rate, most likely because the forward translocation was fast relative to the guanosine addition rate (Figure 4D).

### OZM accelerates the GreA facilitated cleavage of the nascent RNA

We measured the kinetics of GreA-facilitated RNA cleavage of the nascent RNA in the TECs assembled on the nucleic acid scaffolds and extended with the uridine and guanosine (TEC-U-G) or OZM and guanosine (TEC-OZM-G) (Figure 5B). These TECs had 11 bp of RNA:DNA complementarity and could efficiently sample the 1-nt backtracked state. The rate of GreA facilitated RNA cleavage was more than 10-fold higher in the TEC-OZM-G than in the TEC-U-G. OZM could increase the rate of the GreA facilitated RNA cleavage by (i) decreasing the recovery rate from the backtracked state, (ii) increasing the rate of the entry into the backtracked state and (iii) increasing the rate of GreA facilitated RNA cleavage by the backtracked state. We cannot unambiguously differentiate between these not mutually exclusive scenarios but note that attributing the effect of OZM to the facilitated backtracking would be consistent with the effects of OZM incorporation during the processive transcript elongation (see the next two sections). Irrespective of the mechanism by which OZM-promoted GreA facilitated RNA cleavage, the result suggested that the penultimate OZM altered the conformation or dynamics of the nucleic acids within the TEC.

### Incorporation of a single OZM in certain sequence contexts leads to a fractional arrest of processive transcript elongation

**Figure 6.**
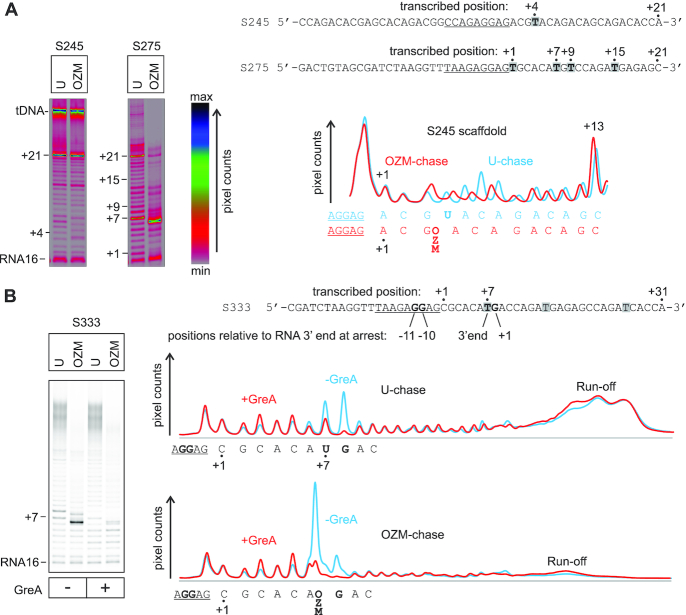
Transcription through a template encoding an OZM-responsive arrest site by *Eco* RNAP. TECs were assembled using the scaffolds shown to the right of the gel panels (only the non-template DNA strands are shown) and chased with 100 μM ATP, CTP, GTP and UTP or OZM-triphosphate for 5 min at 25°C in the presence or absence of 2 μM GreA. The sequence corresponding to the annealing region of the RNA primer is underlined, thymidines in the transcribed region are highlighted. Each assay was performed in triplicate. (**A**) Transcription through two representative short templates. The lane profiles depict the effect of OZM on transcription of the S245 template (left gel panel). Pixel counts were linearly scaled to span 256 gradations within each gel panel. Gels were pseudocolored using the RGB lookup table shown to the right of the gel panels to visualize the low intensity bands. (**B**) Transcription through the simplified and longer derivative of the S275 template (right gel in A) that encodes the OZM-responsive arrest site. The OZM-responsive arrest site at +7 encodes the main determinants of a consensus pause: pyrimidine at the RNA 3′ end, purine at +1 and Gs at −11 and −10, these determinants are shown in bold. Pixel counts were linearly scaled to span the full 8-bit grayscale range. Lane profiles quantified from the gel are presented on the right. The RNA rescued from the arrest site by GreA in OZM-chase does not quantitatively reappear upstream or downstream of the arrest site (see the text for details and possible explanations).

We tested the effect of OZM incorporation on processive transcript elongation by several TECs assembled on the nucleic acid scaffold available in our laboratory. We performed *in vitro* transcription reactions using two different NTP mixtures containing 100 μM ATP, GTP, CTP and UTP (U-chase) or OZM triphosphate (OZM-chase) for 5 min at 25°C. Incorporation of the OZM during transcription of a short template with a single thymidine positioned four base pairs downstream of the RNA primer (Figure 6A, template *S245*) did not measurably impair RNA synthesis as expected from the single nucleotide addition measurement (Figure 5A). The lane profiles of the transcription in the OZM- and U-chases desynchronized at +4 due to the small change in the electrophoretic mobility upon OZM incorporation, but gradually realigned as the transcription progressed further downstream (Figure 6A, profiles to the right of the gel panels). However, transcription of the other short template with thymidines at +1, +7, +9 and +15 (Figure 6A, template S275) in the OZM-chase led to a fractional (∼40%) arrest at +7. At the same time, only ∼20% of the TEC was arrested at +7 in the U-chase. We then redesigned the S275 template by removing the thymidines at +1 and +9 and lengthening the downstream DNA to avoid possible end-of-the-template artifacts. The transcription of the redesign template in the OZM-chase led to a fractional (∼20%) arrest at +7, whereas only ∼6% of the TEC was arrested at +7 in the U-chase (Figure 6B, template S333). These results indicated that the thymidines at +1, +9 and the proximity to the end of the downstream DNA were not essential for the OZM-induced arrest. We then observed that the OZM-responsive arrest site featured the main determinants of a consensus pause: a pyrimidine at the RNA 3′ end, a purine at +1 and guanines at −11 and −10 (96,97). These observations initially suggested that OZM may slow down the recovery from the consensus pause site. However, the addition of 2 μM of GreA completely abolished the OZM induced arrest at +7 suggesting that the arrest was predominantly due to backtracking. While the consensus pause is known to be fractionally backtracked, GreA does not speed up the RNAP escape from the consensus pause site (96,98). We concluded that the pause sequence most likely solely provided time for the TEC to backtrack but was not itself a rate limiting step for the recovery from the OZM-induced arrest.

Perplexingly, GreA prevented arrest at +7 in the OZM-chase but did not increase the amount of the downstream products including the diffuse run-off band (Figure 6B, profiles to the right of the gel panels). At the same time, RNAs rescued from the +7 and +8 arrest sites in the U-chase could be approximately accounted for in the diffuse run-off band. Examination of the full scans of the gels revealed that OZM-chase samples feature an increased amount of RNA that did not enter the gel and remained in the gel wells (Supplementary Figure S6). Notably, we did not encounter problems of this kind with shorter RNAs used in the single nucleotide addition experiments (Figures 2–5). We do not know why long OZM containing RNAs entered the gels poorly. After we recognized the problem, we adjusted our analysis routines as follows. We quantified the fractions of RNAs arrested at the OZM-responsive sites (including the polythymidine tracts, see below) as the ratios of pixel counts of the OZM-induced arrest bands to the pixel counts at-and-above the corresponding sites in the U-chase lanes. This method did not correct for the pipetting errors during the loading of the gels but was immune to the loss of the long RNAs in the OZM-chase samples.

### Incorporation of several successive OZMs leads to transcriptional arrest

**Figure 7.**
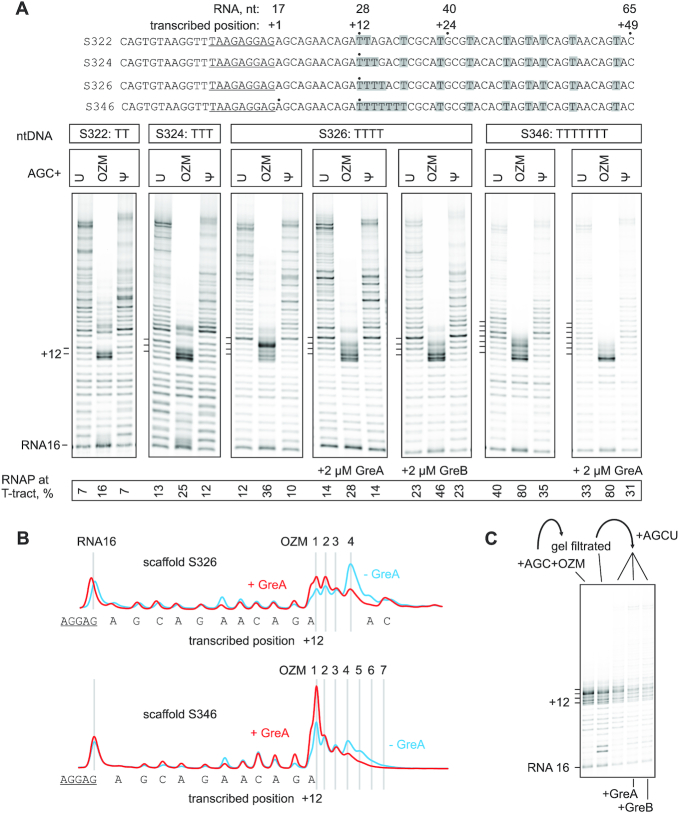
The effect of OZM on transcription through polythymidine sequences by *Eco* RNAP. (**A**) TECs were assembled using the scaffolds shown above the gel panels (only the non-template DNA strands are shown) and chased with 100 μM ATP, CTP, GTP and UTP or OZM triphosphate or ΨTP, for 5 min at 25°C. The sequences corresponding to the annealing region of the RNA primer are underlined. Thymidines in the transcribed region are highlighted. Pixel counts were linearly scaled to span the full 8-bit grayscale range within each gel panel. Each assay was performed in triplicate. (**B**) Lane profiles from gels in (A) depicting the effect of GreA on the transcription of four- and seven-thymidine tracts. (**C**) TECs arrested at the four-thymidine tract (S326 template) were purified by gel-filtration and supplemented with 100 μM NTPs in the absence and presence of 2μM GreA or GreB for 5 min at 25°C.

Considering that a single incorporated OZM had measurable effects on the lateral mobility of the nascent RNA within the TEC as both the 3′ end (Figure 3) and as the penultimate nucleotide (Figure 5B), we reasoned that the incorporation of several consecutive OZM may lead to stronger effects on transcription. We tested the effect of OZM on processive transcription through the template encoding an 11 nt thymidine-free tract followed by two, three, four or seven consecutive thymidines and a relatively thymidine-rich sequence further downstream (Figure 7A). We performed *in vitro* transcription reactions using three different NTP mixtures containing 100 μM ATP, GTP, CTP and UTP (U-chase) or OZM triphosphate (OZM-chase) or ΨTP (Ψ-chase) for 5 min at 25°C. OZM did not affect transcription through the thymidine-free tract as expected but arrested a significant fraction of RNAP at the polythymidine sequences. Thus, approximately 2-fold more RNAP was retained at the polythymidine tracts following the 5-min incubation in the OZM-chase compared with the U-chase (Figure 7A). The arrested RNAs corresponded to the end of the four-thymidine tract and to the beginning of the two-, three and seven-thymidine tracts. In the presence of Gre RNA cleavage factors the arrested RNAs predominantly corresponded to the beginning of the four-thymidine tract suggesting that the arrested TECs were predominantly backtracked (Figure 7A and B). GreA failed to release the RNAPs arrested at the four- and seven-thymidine tracts in the OZM-chase (Figure 7A and B), but reactivated the arrested TECs and allowed the synthesis of the full-length transcripts if the OZM-chase was removed by gel filtration and replaced by a U-chase (Figure 7C). These results suggest that the rate of backtracking greatly exceeded the rate of the nucleotide addition following the incorporation of several consecutive OZMs and the TECs failed to make through the polythymidine tracts despite being continuously rescued by the Gre facilitated cleavage of the nascent RNAs. While the loss of long OZM-containing RNAs complicated the estimation of the read-through the arrest sites (see the previous section), the effect of GreA was evidently different at the arrest sites caused by a single (Figure 6) and multiple (Figure 7) OZMs: GreA alleviated the arrest at the former but not at the latter sites.

**Figure 8.**
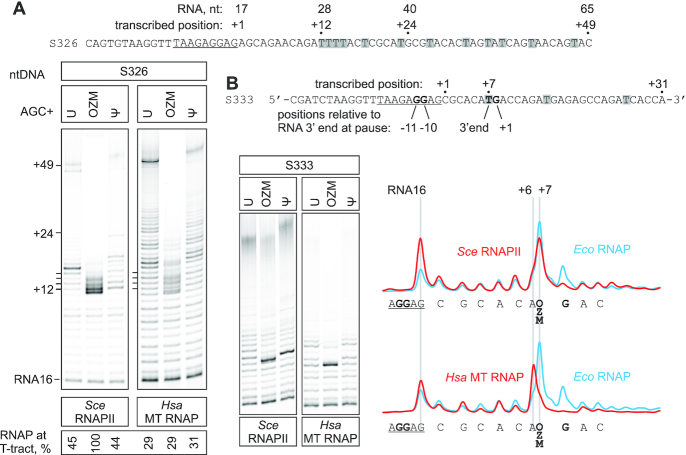
The effect of OZM on processive transcription by *Sce* RNAPII and *Hsa* MT RNAP. TECs were assembled using the scaffolds shown above the gel panels (only the non-template DNA strands are shown) and chased with 100 μM ATP, CTP, GTP and UTP or OZM triphosphate or ΨTP, for 5 min at 25°C. The sequences corresponding to the annealing region of the RNA primer are underlined. Thymidines in the transcribed region are highlighted. Pixel counts were linearly scaled to span the full 8-bit grayscale range within each gel panel. Each assay was performed in triplicate. (**A**) Transcription through the four-thymidine tract. (**B**) Transcription through the OZM-responsive arrest site. OZM lane traces are shown to the right, the *Eco* RNAP trace was quantified from the gel in Figure 6B.

*Sce* RNAPII was arrested at the four-thymidine tract and the single thymidine OZM-responsive arrest site similarly to the *Eco* RNAP (Figure 8) except that most of the arrested RNA corresponded to the beginning of the four-thymidine tract in the case of *Sce* RNAPII. In contrast, no significant accumulation of the arrested RNAs within the four-thymidine tract was evident during transcription by *Hsa* MT RNAP (∼30% of RNAs correspond to the polythymidine tract in U-, OZM- and Ψ-chase lanes in Figure 8A). While the intensity of long transcripts was significantly lower in the OZM-chase than in the U-chase, the difference may be in part or entirely attributable to the loss of long OZM-containing RNAs rather than the bona fide inhibition of transcription. Similarly, while a fraction of MT RNAP was arrested near the OZM-responsive site in the OZM-chase, the arrested RNA corresponded to the sequence position preceding the site of the OZM incorporation (Figure 8B, lower trace). The latter result suggests a different mode of inhibition or, alternatively, can be explained by a high exonuclease or pyrophosphorolysis activity of MT RNAP at the arrest site. We did not investigate this phenomenon further. Overall, our data indicated that the OZM incorporation had a similar inhibitory effect on the multisubunit RNAPs from bacteria and yeast, whereas the single-subunit mitochondrial RNAP responded differently to OZM incorporation.

## DISCUSSION

In this study, we characterized the inhibitory activity of the C-nucleoside antibiotic OZM on transcription by multisubunit RNAPs from *E. coli* and *S. cerevisiae* and a single subunit RNAP from human mitochondria. We show that the OZM triphosphate is a good substrate for *Eco* RNAP and is also efficiently incorporated into RNA by *Sce* RNAPII and *Hsa* MT RNAP. A detailed analysis of the translocation equilibrium suggested that the 3′ terminal and the penultimate OZM increased the lateral mobility of the nascent RNA within the RNAP and favored backward translocation by one or more registers. However, the above effects were apparently benign for the processive transcription of some sequences and the incorporated OZM was rapidly extended by the next nucleotide (Figures 5A and 6A). In certain sequence contexts, incorporation of OZM arrested a significant fraction of the RNAP. One example was the consensus-pause-like sequence (96,97), where the arrest could be alleviated by the Gre-facilitated cleavage of the nascent RNA. The other examples were the polythymidine sequences, where GreA and GreB shortened the arrested RNAs but failed to release the RNAP from the arrest region. However, the RNAP arrested at the polythymidine tracts was efficiently recovered by Gre factors if the OZM triphosphate was removed from the reaction medium. Overall, our data suggested that OZM arrested transcription exclusively by promoting backtracking. While the consensus-pause-like sequence was involved in arresting RNAP in one case, the alleviation of the arrest by GreA suggested that the escape from the pause was not rate limiting (96,98), and that the pause merely provided time for entry into the backtracked state by a fraction of the transcribing RNAP. Noteworthy, a synthetic pyrazine-carboxamide nucleotide analog T-1106 has been recently suggested to inhibit viral RNA-dependent RNA polymerase by promoting backtracking (99).

Our data also suggest that the incorporation of OZM significantly alters the properties of RNAs compared with the uridine containing RNAs. First, the incorporation of OZM into RNA resulted in a very small change in the electrophoretic mobility of the RNA compared to that upon uridine or pseudouridine incorporation. Second, long RNAs with incorporated OZM entered the denaturing PAGE gels poorly for some unknown reason. Third, from a purely theoretical standpoint, the free electron pair of the OZM ring oxygen and its partial resonance to the carbonyl group at C2 may lead to deviations in the secondary and tertiary structures compared to the native, uridine-containing RNAs. Most importantly, both the transcriptional effects of OZM and the unusual properties of OZM-containing RNA could be attributed to an oxygen atom in the position corresponding to the carbon five of the uracil ring rather than to the C-glycosidic bond. Thus, the ubiquitous C-nucleoside pseudouridine did not cause transcriptional arrest at the OZM-responsive sites and the pseudouridine-containing RNAs did not display the anomalous properties of the OZM-containing RNAs.

We argue that OZM functions as a Trojan horse substrate. OZM enters the target organism in nucleoside form, becomes phosphorylated by the recipient’s kinases (20) and competes with UTP for incorporation into RNA. It is commonly accepted that selection of the cognate NTPs by the RNAPs relies on (i) proper Watson–Crick bonding with the acceptor template DNA base in the active site, (ii) the recognition of the 2′ and 3′ OH of the ribose and (iii) the recognition of a triphosphate moiety. The OZM triphosphate features all these determinants and is therefore largely indistinguishable from UTP as the RNAP substrate. However, upon the incorporation of nucleotides into RNA, the stacking interactions between the neighboring bases start playing a very significant role in determining the shape and the flexibility of the nucleic acid duplexes. For example, polythymidine/polyadenine sequences adopt an unusual helical conformation with a high propeller twist and bifurcating hydrogen bonds to maximize the stacking interactions between the nucleobases (100,101). OZM has an oxygen atom in the position corresponding to carbon five of the uracil ring and therefore may stack differently than UTP with the neighboring bases after its incorporation into RNA. Notably, none of the four native nucleobases contains oxygen atoms within the ring structures. The altered stacking can plausibly modify the RNA:DNA hybrid structure, flexibility and interactions with RNAP thereby leading to backtracking and transcriptional arrest.

In this study, we assessed the effects of OZM on transcription *in vitro* and our data suggest that at a high OZM/U ratio *in vivo*, OZM should primarily act as a transcription inhibitor by arresting RNAP at the frequently encountered sequences such as three-four-thymidine tracts. However, at a low OZM/U ratio in the target cell, the probability of the incorporation of several successive OZMs is low and OZM may be permissive for transcription. Under these conditions, the post-transcriptional effect, such as altered RNA secondary structures or interference with translation may potentially contribute significantly to the inhibition of the cellular functions. For example, the targeted pseudouridinylation of the mRNAs has been shown to cause the read through of stop codons by the ribosome (7). Accordingly, a full understanding of the OZM mechanism of action awaits a thorough evaluation of its effects on the folding of structural RNAs, translation of RNA into protein and studies of the relative impact of a sublethal OZM concentration on transcription and post-transcriptional processes *in vivo*.

## Supplementary Material

gkz782_Supplemental_File

## References

[1] Wallace S.S. Base excision repair: a critical player in many games. DNA Repair. 2014; 19:14–26. 24780558 10.1016/j.dnarep.2014.03.030 PMC4100245

[2] Prakash A., Doublié S. Base excision repair in the Mitochondria. J. Cell Biochem. 2015; 116:1490–1499. 25754732 10.1002/jcb.25103 PMC4546830

[3] Gruber C.C., Walker G.C. Incomplete base excision repair contributes to cell death from antibiotics and other stresses. DNA Repair. 2018; 71:108–117. 30181041 10.1016/j.dnarep.2018.08.014 PMC6442677

[4] Kren V., Martínková L. Glycosides in medicine: ‘The role of glycosidic residue in biological activity’. Curr. Med. Chem. 2001; 8:1303–1328. 11562268 10.2174/0929867013372193

[5] Weatherman R. V, Mortell K.H., Chervenak M., Kiessling L.L., Toone E.J. Specificity of C-glycoside complexation by mannose/glucose specific lectins. Biochemistry. 1996; 35:3619–3624. 8639514 10.1021/bi951916z

[6] Charette M., Gray M.W. Pseudouridine in RNA: what, where, how, and why. IUBMB Life. 2000; 49:341–351. 10902565 10.1080/152165400410182

[7] Karijolich J., Yu Y.-T. Converting nonsense codons into sense codons by targeted pseudouridylation. Nature. 2011; 474:395–398. 21677757 10.1038/nature10165 PMC3381908

[8] Carlile T.M., Rojas-Duran M.F., Zinshteyn B., Shin H., Bartoli K.M., Gilbert W. V. Pseudouridine profiling reveals regulated mRNA pseudouridylation in yeast and human cells. Nature. 2014; 515:143–146. 25192136 10.1038/nature13802 PMC4224642

[9] Bililign T., Griffith B.R., Thorson J.S. Structure, activity, synthesis and biosynthesis of aryl-C-glycosides. Nat. Prod. Rep. 2005; 22:742–760. 16311633 10.1039/b407364a

[10] Hultin P.G. Bioactive C-glycosides from bacterial secondary metabolism. Curr. Top. Med. Chem. 2005; 5:1299–1331. 16305533 10.2174/156802605774643015

[11] Sosio M., Gaspari E., Iorio M., Pessina S., Medema M.H., Bernasconi A., Simone M., Maffioli S.I., Ebright R.H., Donadio S. Analysis of the pseudouridimycin biosynthetic pathway provides insights into the formation of C-nucleoside antibiotics. Cell Chem. Biol. 2018; 25:540–549. 29551347 10.1016/j.chembiol.2018.02.008 PMC5959762

[12] Wang S.-A., Ko Y., Zeng J., Geng Y., Ren D., Ogasawara Y., Irani S., Zhang Y., Liu H. Identification of the formycin A biosynthetic gene cluster from *Streptomyces kaniharaensis* illustrates the interplay between biological pyrazolopyrimidine formation and *de Novo* purine biosynthesis. J. Am. Chem. Soc. 2019; 141:6127–6131. 30942582 10.1021/jacs.9b00241 PMC6612245

[13] Palmu K., Rosenqvist P., Thapa K., Ilina Y., Siitonen V., Baral B., Mäkinen J., Belogurov G., Virta P., Niemi J. et al. Discovery of the showdomycin gene cluster from streptomyces showdoensis ATCC 15227 yields insight into the biosynthetic logic of C-Nucleoside antibiotics. ACS Chem. Biol. 2017; 12:1472–1477. 28418235 10.1021/acschembio.7b00078

[14] Hong H., Samborskyy M., Zhou Y., Leadlay P.F. C-Nucleoside formation in the biosynthesis of the antifungal malayamycin A. Cell Chem. Biol. 2019; 26:493–501. 30713097 10.1016/j.chembiol.2018.12.004

[15] Haneishi T., Okazaki T., Tadashi H., Chihiro T., Masako N., Atsushi N., Seki I., Arai M. Oxazinomycin, A new Carbo-linked nucleoside antibiotic. J. Antibiot. 1971; 24:797–799. 5140526 10.7164/antibiotics.24.797

[16] Kusakabe Y., Nagatsu J., Shibuya M., Kawaguchi O., Hirose C., Shirato S. Minimycin, a new antibiotic. J. Antibiot. 1972; 25:44–47. 5010645 10.7164/antibiotics.25.44

[17] Sasaki K., Kusakabe Y., Esumi S. The structure of minimycin, A novel Carbon-linked nucleoside antibiotic related to b-Pseudouridine. J. Antibiot. 1972; 25:151–154. 5034811 10.7164/antibiotics.25.151

[18] Tymiak A.A., Culver C.A., Goodman J.F., Seiner V.S., Sykes R.B. Oxazinomycin produced by a Pseudomonas species. J. Antibiot. 1984; 37:416–418. 6725147 10.7164/antibiotics.37.416

[19] De Bernardo S., Weigele M. Synthesis of oxazinomycin (Minimycin). J. Org. Chem. 1977; 42:109–112. 830847 10.1021/jo00421a021

[20] Deville-Bonne D., El Amri C., Meyer P., Chen Y., Agrofoglio L.A., Janin J. Human and viral nucleoside/nucleotide kinases involved in antiviral drug activation: Structural and catalytic properties. Antiviral Res. 2010; 86:101–120. 20417378 10.1016/j.antiviral.2010.02.001

[21] Werner F., Grohmann D. Evolution of multisubunit RNA polymerases in the three domains of life. Nat. Rev. Microbiol. 2011; 9:85–98. 21233849 10.1038/nrmicro2507

[22] Cermakian N., Ikeda T.M., Miramontes P., Lang B.F., Gray M.W., Cedergren R. On the evolution of the single-subunit RNA polymerases. J. Mol. Evol. 1997; 45:671–681. 9419244 10.1007/pl00006271

[23] Belogurov G.A., Artsimovitch I. Regulation of transcript elongation. Annu. Rev. Microbiol. 2015; 69:49–69. 26132790 10.1146/annurev-micro-091014-104047 PMC4674076

[24] Hillen H.S., Temiakov D., Cramer P. Structural basis of mitochondrial transcription. Nat. Struct. Mol. Biol. 2018; 25:754–765. 30190598 10.1038/s41594-018-0122-9 PMC6583890

[25] Guajardo R., Sousa R. A model for the mechanism of polymerase translocation. J. Mol. Biol. 1997; 265:8–19. 8995520 10.1006/jmbi.1996.0707

[26] Bai L., Shundrovsky A., Wang M.D. Sequence-dependent kinetic model for transcription elongation by RNA polymerase. J. Mol. Biol. 2004; 344:335–349. 15522289 10.1016/j.jmb.2004.08.107

[27] Bar-Nahum G., Epshtein V., Ruckenstein A.E., Rafikov R., Mustaev A., Nudler E. A ratchet mechanism of transcription elongation and its control. Cell. 2005; 120:183–193. 15680325 10.1016/j.cell.2004.11.045

[28] Abbondanzieri E.A., Greenleaf W.J., Shaevitz J.W., Landick R., Block S.M. Direct observation of base-pair stepping by RNA polymerase. Nature. 2005; 438:460–465. 16284617 10.1038/nature04268 PMC1356566

[29] Ó Maoiléidigh D., Tadigotla V.R., Nudler E., Ruckenstein A.E. A unified model of transcription elongation: what have we learned from single-molecule experiments. Biophys. J. 2011; 100:1157–1166. 21354388 10.1016/j.bpj.2010.12.3734 PMC3043204

[30] Malinen A.M., Turtola M., Parthiban M., Vainonen L., Johnson M.S., Belogurov G.A. Active site opening and closure control translocation of multisubunit RNA polymerase. Nucleic Acids Res. 2012; 40:7442–7451. 22570421 10.1093/nar/gks383 PMC3424550

[31] Nedialkov Y.A., Nudler E., Burton Z.F. RNA polymerase stalls in a post-translocated register and can hyper-translocate. Transcription. 2012; 3:260–269. 23132506 10.4161/trns.22307 PMC3632624

[32] Kireeva M., Trang C., Matevosyan G., Turek-Herman J., Chasov V., Lubkowska L., Kashlev M. RNA–DNA and DNA–DNA base-pairing at the upstream edge of the transcription bubble regulate translocation of RNA polymerase and transcription rate. Nucleic Acids Res. 2018; 46:5764–5775. 29771376 10.1093/nar/gky393 PMC6009650

[33] Komissarova N., Kashlev M. Transcriptional arrest: Escherichia coli RNA polymerase translocates backward, leaving the 3′ end of the RNA intact and extruded. Proc. Natl. Acad. Sci. U.S.A. 1997; 94:1755–1760. 9050851 10.1073/pnas.94.5.1755 PMC19989

[34] Nudler E., Mustaev A., Lukhtanov E., Goldfarb A. The RNA-DNA hybrid maintains the register of transcription by preventing backtracking of RNA polymerase. Cell. 1997; 89:33–41. 9094712 10.1016/s0092-8674(00)80180-4

[35] Artsimovitch I., Landick R. Pausing by bacterial RNA polymerase is mediated by mechanistically distinct classes of signals. Proc. Natl. Acad. Sci. U.S.A. 2000; 97:7090–7095. 10860976 10.1073/pnas.97.13.7090 PMC16504

[36] Epshtein V., Toulme F., Rahmouni R., Borukhov S., Nudler E. Transcription through the roadblocks: the role of RNA polymerase cooperation. EMBO J. 2003; 22:4719–4727. 12970184 10.1093/emboj/cdg452 PMC212720

[37] Dangkulwanich M., Ishibashi T., Liu S., Kireeva M.L., Lubkowska L., Kashlev M., Bustamante C.J. Complete dissection of transcription elongation reveals slow translocation of RNA polymerase II in a linear ratchet mechanism. Elife. 2013; 2:e00971. 24066225 10.7554/eLife.00971 PMC3778554

[38] Kotlajich M. V., Hron D.R., Boudreau B.A., Sun Z., Lyubchenko Y.L., Landick R. Bridged filaments of histone-like nucleoid structuring protein pause RNA polymerase and aid termination in bacteria. Elife. 2015; 4:e04970. 10.7554/eLife.04970 PMC4337669 25594903

[39] Charlet-Berguerand N., Feuerhahn S., Kong S.E., Ziserman H., Conaway J.W., Conaway R., Egly J.M. RNA polymerase II bypass of oxidative DNA damage is regulated by transcription elongation factors. EMBO J. 2006; 25:5481–5491. 17110932 10.1038/sj.emboj.7601403 PMC1679758

[40] Xu L., Wang W., Wu J., Shin J.H., Wang P., Unarta I.C., Chong J., Wang Y., Wang D. Mechanism of DNA alkylation-induced transcriptional stalling, lesion bypass, and mutagenesis. Proc. Natl. Acad. Sci. U.S.A. 2017; 114:E7082–E7091. 28784758 10.1073/pnas.1708748114 PMC5576830

[41] Da L.T., Pardo-Avila F., Xu L., Silva D.A., Zhang L., Gao X., Wang D., Huang X. Bridge helix bending promotes RNA polymerase II backtracking through a critical and conserved threonine residue. Nat. Commun. 2016; 7:11244. 27091704 10.1038/ncomms11244 PMC4838855

[42] Wang D., Levitt M., Kornberg R.D., Bushnell D.A., Huang X., Westover K.D., Levitt M., Kornberg R.D. Structural basis of transcription: backtracked RNA polymerase II at 3.4 angstrom resolution. Science. 2009; 324:1203–1206. 19478184 10.1126/science.1168729 PMC2718261

[43] Sekine S., Murayama Y., Svetlov V., Nudler E., Yokoyama S. The ratcheted and ratchetable structural states of RNA polymerase underlie multiple transcriptional functions. Mol. Cell. 2015; 57:408–421. 25601758 10.1016/j.molcel.2014.12.014

[44] Sosunova E., Sosunov V., Epshtein V., Nikiforov V., Mustaev A. Control of transcriptional fidelity by active center tuning as derived from RNA polymerase endonuclease reaction. J. Biol. Chem. 2013; 288:6688–6703. 23283976 10.1074/jbc.M112.424002 PMC5396497

[45] Turtola M., Mäkinen J.J., Belogurov G.A. Active site closure stabilizes the backtracked state of RNA polymerase. Nucleic Acids Res. 2018; 46:10870–10887. 30256972 10.1093/nar/gky883 PMC6237748

[46] Cheung A.C.M., Cramer P. Structural basis of RNA polymerase II backtracking, arrest and reactivation. Nature. 2011; 471:249–253. 21346759 10.1038/nature09785

[47] Abdelkareem M., Saint-André C., Takacs M., Papai G., Crucifix C., Guo X., Ortiz J., Weixlbaumer A. Structural basis of transcription: RNA polymerase backtracking and its reactivation. Mol. Cell. 2019; 75:298–309. 31103420 10.1016/j.molcel.2019.04.029 PMC7611809

[48] Orlova M., Newlandst J., Dast A., Goldfarb A., Borukhov S. Intrinsic transcript cleavage activity of RNA polymerase. Proc. Natl. Acad. Sci. U.S.A. 1995; 92:4596–4600. 7538676 10.1073/pnas.92.10.4596 PMC41991

[49] Borukhov S., Sagitov V., Goldfarb A. Transcript cleavage factors from E. coli. Cell. 1993; 72:459–466. 8431948 10.1016/0092-8674(93)90121-6

[50] Laptenko O., Lee J., Lomakin I., Borukhov S. Transcript cleavage factors GreA and GreB act as transient catalytic components of RNA polymerase. EMBO J. 2003; 22:6322–6334. 14633991 10.1093/emboj/cdg610 PMC291851

[51] Hausner W., Lange U., Musfeldt M. Transcription factor S, a cleavage induction factor of the archaeal RNA polymerase. J. Biol. Chem. 2000; 275:12393–12399. 10777522 10.1074/jbc.275.17.12393

[52] Fouqueau T., Blombach F., Hartman R., Cheung A.C.M., Young M.J., Werner F. The transcript cleavage factor paralogue TFS4 is a potent RNA polymerase inhibitor. Nat. Commun. 2017; 8:1914. 29203770 10.1038/s41467-017-02081-3 PMC5715097

[53] Izban M.G., Luse D.S. The RNA polymerase II ternary complex cleaves the nascent transcript in a 3′—-5′ direction in the presence of elongation factor SII. Genes Dev. 1992; 6:1342–1356. 1378419 10.1101/gad.6.7.1342

[54] Reines D. Elongation factor-dependent transcript shortening by template-engaged RNA polymerase II. J. Biol. Chem. 1992; 267:3795–3800. 1371280 PMC3373963

[55] Ruan W., Lehmann E., Thomm M., Kostrewa D., Cramer P. Evolution of two modes of intrinsic RNA polymerase transcript cleavage. J. Biol. Chem. 2011; 286:18701–18707. 21454497 10.1074/jbc.M111.222273 PMC3099687

[56] Zamft B., Bintu L., Ishibashi T., Bustamante C. Nascent RNA structure modulates the transcriptional dynamics of RNA polymerases. Proc. Natl. Acad. Sci. U.S.A. 2012; 109:8948–8953. 22615360 10.1073/pnas.1205063109 PMC3384149

[57] Da L.-T., E C., Shuai Y., Wu S., Su X.-D., Yu J. T7 RNA polymerase translocation is facilitated by a helix opening on the fingers domain that may also prevent backtracking. Nucleic Acids Res. 2017; 45:7909–7921. 28575393 10.1093/nar/gkx495 PMC5737862

[58] Ho M.X., Hudson B.P., Das K., Arnold E., Ebright R.H. Structures of RNA polymerase-antibiotic complexes. Curr. Opin. Struct. Biol. 2009; 19:715–723. 19926275 10.1016/j.sbi.2009.10.010 PMC2950656

[59] Brodolin K. Antibiotics targeting bacterial RNA polymerase. Antibiotics. 2013; Weinheim Wiley-VCH Verlag GmbH & Co. KGaA 299–321.

[60] Campbell E.A., Korzheva N., Mustaev A., Murakami K., Nair S., Goldfarb A., Darst S.A. Structural mechanism for rifampicin inhibition of bacterial RNA polymerase. Cell. 2001; 104:901–912. 11290327 10.1016/s0092-8674(01)00286-0

[61] Ma C., Yang X., Lewis P.J. Bacterial transcription as a target for antibacterial drug development. Microbiol. Mol. Biol. Rev. 2016; 80:139–160. 26764017 10.1128/MMBR.00055-15 PMC4771368

[62] Artsimovitch I., Vassylyeva M.N., Svetlov D., Svetlov V., Perederina A., Igarashi N., Matsugaki N., Wakatsuki S., Tahirov T.H., Vassylyev D.G. Allosteric modulation of the RNA polymerase catalytic reaction is an essential component of transcription control by rifamycins. Cell. 2005; 122:351–363. 16096056 10.1016/j.cell.2005.07.014

[63] Molodtsov V., Scharf N.T., Stefan M.A., Garcia G.A., Murakami K.S. Structural basis for rifamycin resistance of bacterial RNA polymerase by the three most clinically important RpoB mutations found in *Mycobacterium tuberculosis*. Mol. Microbiol. 2017; 103:1034–1045. 28009073 10.1111/mmi.13606 PMC5344776

[64] Mosaei H., Molodtsov V., Kepplinger B., Harbottle J., Moon C.W., Jeeves R.E., Ceccaroni L., Shin Y., Morton-Laing S., Marrs E.C.L. et al. Mode of action of kanglemycin a, an ansamycin natural product that is active against rifampicin-resistant mycobacterium tuberculosis. Mol. Cell. 2018; 72:263–274. 30244835 10.1016/j.molcel.2018.08.028 PMC6202310

[65] Temiakov D., Zenkin N., Vassylyeva M.N., Perederina A., Tahirov T.H., Kashkina E., Savkina M., Zorov S., Nikiforov V., Igarashi N. et al. Structural basis of transcription inhibition by antibiotic streptolydigin. Mol. Cell. 2005; 19:655–666. 16167380 10.1016/j.molcel.2005.07.020

[66] Tuske S., Sarafianos S.G., Wang X., Hudson B., Sineva E., Mukhopadhyay J., Birktoft J.J., Leroy O., Ismail S., Clark A.D. Jr et al. Inhibition of bacterial RNA polymerase by streptolydigin: stabilization of a straight-bridge-helix active-center conformation. Cell. 2005; 122:541–552. 16122422 10.1016/j.cell.2005.07.017 PMC2754413

[67] Pavlova O., Mukhopadhyay J., Sineva E., Ebright R.H., Severinov K. Systematic structure-activity analysis of microcin J25. J. Biol. Chem. 2008; 283:25589–25595. 18632663 10.1074/jbc.M803995200 PMC2533079

[68] Mukhopadhyay J., Sineva E., Knight J., Levy R.M., Ebright R.H. Antibacterial peptide microcin J25 inhibits transcription by binding within and obstructing the RNA polymerase secondary channel. Mol. Cell. 2004; 14:739–751. 15200952 10.1016/j.molcel.2004.06.010 PMC2754415

[69] Belogurov G.A., Vassylyeva M.N., Sevostyanova A., Appleman J.R., Xiang A.X., Lira R., Webber S.E., Klyuyev S., Nudler E., Artsimovitch I. et al. Transcription inactivation through local refolding of the RNA polymerase structure. Nature. 2009; 457:332–335. 18946472 10.1038/nature07510 PMC2628454

[70] Mukhopadhyay J., Das K., Ismail S., Koppstein D., Jang M., Hudson B., Sarafianos S., Tuske S., Patel J., Jansen R. et al. The RNA polymerase ‘switch region’ is a target for inhibitors. Cell. 2008; 135:295–307. 18957204 10.1016/j.cell.2008.09.033 PMC2580802

[71] Degen D., Feng Y., Zhang Y., Ebright K.Y., Ebright Y.W., Gigliotti M., Vahedian-Movahed H., Mandal S., Talaue M., Connell N. et al. Transcription inhibition by the depsipeptide antibiotic salinamide A. Elife. 2014; 3:e02451. 24843001 10.7554/eLife.02451 PMC4029172

[72] Zhang Y., Degen D., Ho M.X., Sineva E., Ebright K.Y., Ebright Y.W., Mekler V., Vahedian-Movahed H., Feng Y., Yin R. et al. GE23077 binds to the RNA polymerase ‘i’ and ‘i+1’ sites and prevents the binding of initiating nucleotides. Elife. 2014; 3:e02450. 24755292 10.7554/eLife.02450 PMC3994528

[73] Bae B., Nayak D., Ray A., Mustaev A., Landick R., Darst S.A. CBR antimicrobials inhibit RNA polymerase via at least two bridge-helix cap-mediated effects on nucleotide addition. Proc. Natl. Acad. Sci. U.S.A. 2015; 112:E4178–E4187. 26195788 10.1073/pnas.1502368112 PMC4534225

[74] Feng Y., Degen D., Wang X., Gigliotti M., Liu S., Zhang Y., Das D., Michalchuk T., Ebright Y.W., Talaue M. et al. Structural basis of transcription inhibition by CBR hydroxamidines and CBR pyrazoles. Structure. 2015; 23:1470–1481. 26190576 10.1016/j.str.2015.06.009 PMC4526357

[75] Artsimovitch I., Chu C., Lynch A.S., Landick R. A new class of bacterial RNA polymerase inhibitor affects nucleotide addition. Science. 2003; 302:650–654. 14576436 10.1126/science.1087526

[76] Lin W., Das K., Degen D., Mazumder A., Duchi D., Wang D., Ebright Y.W., Ebright R.Y., Sineva E., Gigliotti M. et al. Structural basis of transcription inhibition by fidaxomicin (Lipiarmycin A3). Mol. Cell. 2018; 70:60–71. 29606590 10.1016/j.molcel.2018.02.026 PMC6205224

[77] Boyaci H., Chen J., Lilic M., Palka M., Mooney R.A., Landick R., Darst S.A., Campbell E.A. Fidaxomicin jams mycobacterium tuberculosis RNA polymerase motions needed for initiation via RBPA contacts. Elife. 2018; 7:34823. 10.7554/eLife.34823 PMC5837556 29480804

[78] Tupin A., Gualtieri M., Leonetti J.-P.P., Brodolin K. The transcription inhibitor lipiarmycin blocks DNA fitting into the RNA polymerase catalytic site. EMBO J. 2010; 29:2527–2537. 20562828 10.1038/emboj.2010.135 PMC2928680

[79] Maffioli S.I., Zhang Y., Degen D., Carzaniga T., Del Gatto G., Serina S., Monciardini P., Mazzetti C., Guglierame P., Candiani G. et al. Antibacterial nucleoside-analog inhibitor of bacterial RNA polymerase. Cell. 2017; 169:1240–1248. 28622509 10.1016/j.cell.2017.05.042 PMC5542026

[80] Svetlov V., Artsimovitch I. Purification of bacterial RNA polymerase: tools and protocols. Methods Mol. Biol. 2015; 1276:13–29. 25665556 10.1007/978-1-4939-2392-2_2 PMC4324551

[81] Kireeva M.L., Lubkowska L., Komissarova N., Kashlev M. Assays and affinity purification of biotinylated and nonbiotinylated forms of double-tagged core RNA polymerase II from Saccharomyces cerevisiae. Methods Enzymol. 2003; 370:138–155. 14712640 10.1016/S0076-6879(03)70012-3

[82] Sydow J.F., Brueckner F., Cheung A.C., Damsma G.E., Dengl S., Lehmann E., Vassylyev D., Cramer P. Structural basis of transcription: mismatch-specific fidelity mechanisms and paused RNA polymerase II with frayed RNA. Mol. Cell. 2009; 34:710–721. 19560423 10.1016/j.molcel.2009.06.002

[83] Ringel R., Sologub M., Morozov Y.I., Litonin D., Cramer P., Temiakov D. Structure of human mitochondrial RNA polymerase. Nature. 2011; 478:269–273. 21947009 10.1038/nature10435

[84] Perederina A.A., Vassylyeva M.N., Berezin I.A., Svetlov V., Artsimovitch I., Vassylyev D.G. Cloning, expression, purification, crystallization and initial crystallographic analysis of transcription elongation factors GreB from Escherichia coli and Gfh1 from Thermus thermophilus. Acta Crystallogr. Sect. F Struct. Biol. Cryst. Commun. 2006; 62:44–46. 10.1107/S1744309105040297 PMC1401493 16511259

[85] Komissarova N., Kireeva M.L., Becker J., Sidorenkov I., Kashlev M. Engineering of elongation complexes of bacterial and yeast RNA polymerases. Methods Enzymol. 2003; 371:233–251. 14712704 10.1016/S0076-6879(03)71017-9

[86] Abramoff M.D., Magalhaes P.J., Ram S.J. Image processing with ImageJ. Biophotonics Int. 2004; 11:36–42.

[87] Johnson K.A. Fitting enzyme kinetic data with KinTek Global Kinetic Explorer. Methods Enzymol. 2009; 467:601–626. 19897109 10.1016/S0076-6879(09)67023-3

[88] Malinen A.M., NandyMazumdar M., Turtola M., Malmi H., Grocholski T., Artsimovitch I., Belogurov G.A. CBR antimicrobials alter coupling between the bridge helix and the β subunit in RNA polymerase. Nat. Commun. 2014; 5:3408. 24598909 10.1038/ncomms4408 PMC3959191

[89] Malinen A.M., Turtola M., Belogurov G.A. Monitoring translocation of multisubunit RNA polymerase along the DNA with fluorescent base analogues. Methods Mol. Biol. 2015; 1276:31–51. 25665557 10.1007/978-1-4939-2392-2_3

[90] Kireeva M.L., Nedialkov Y.A., Cremona G.H., Purtov Y.A., Lubkowska L., Malagon F., Burton Z.F., Strathern J.N., Kashlev M. Transient reversal of RNA polymerase II active site closing controls fidelity of transcription elongation. Mol. Cell. 2008; 30:557–566. 18538654 10.1016/j.molcel.2008.04.017 PMC7243879

[91] Turtola M., Belogurov G.A. NusG inhibits RNA polymerase backtracking by stabilizing the minimal transcription bubble. Elife. 2016; 5:e18096. 27697152 10.7554/eLife.18096 PMC5100998

[92] Yoshikawa M., Kato T., Takenishi T. A novel method for phosphorylation of nucleosides to 5′-nucleotides. Tetrahedron Lett. 1967; 50:5065–5068. 6081184 10.1016/s0040-4039(01)89915-9

[93] Hawkins M.E. Synthesis, purification and sample experiment for fluorescent pteridine-containing DNA: tools for studying DNA interactive systems. Nat. Protoc. 2007; 2:1013–1021. 17446875 10.1038/nprot.2007.150

[94] Martin C.T., Újvári A., Liu C., Ujvari A., Liu C. Evaluation of fluorescence spectroscopy methods for mapping melted regions of DNA along the transcription pathway. Methods Enzymol. 2003; 371:13–33. 14712689 10.1016/S0076-6879(03)71002-7

[95] Basu R.S., Warner B.A., Molodtsov V., Pupov D., Esyunina D., Fernández-Tornero C., Kulbachinskiy A., Murakami K.S. Structural basis of transcription initiation by bacterial RNA polymerase holoenzyme. J. Biol. Chem. 2014; 289:24549–24559. 24973216 10.1074/jbc.M114.584037 PMC4148879

[96] Larson M.H., Mooney R.A., Peters J.M., Windgassen T., Nayak D., Gross C.A., Block S.M., Greenleaf W.J., Landick R., Weissman J.S. A pause sequence enriched at translation start sites drives transcription dynamics in vivo. Science. 2014; 344:1042–1047. 24789973 10.1126/science.1251871 PMC4108260

[97] Vvedenskaya I.O., Vahedian-Movahed H., Bird J.G., Knoblauch J.G., Goldman S.R., Zhang Y., Ebright R.H., Nickels B.E. Interactions between RNA polymerase and the ‘core recognition element’ counteract pausing. Science. 2014; 344:1285–1289. 24926020 10.1126/science.1253458 PMC4277259

[98] Saba J., Chua X.Y., Mishanina T. V, Nayak D., Windgassen T.A., Mooney R.A., Landick R. The elemental mechanism of transcriptional pausing. Elife. 2019; 8:40981. 10.7554/eLife.40981 PMC6336406 30618376

[99] Dulin D., Arnold J.J., van Laar T., Oh H.-S., Lee C., Perkins A.L., Harki D.A., Depken M., Cameron C.E., Dekker N.H. Signatures of nucleotide analog incorporation by an RNA-dependent RNA polymerase revealed using high-throughput magnetic tweezers. Cell Rep. 2017; 21:1063–1076. 29069588 10.1016/j.celrep.2017.10.005 PMC5670035

[100] Coll M., Frederick C.A., Wang A.H., Rich A. A bifurcated hydrogen-bonded conformation in the d(A.T) base pairs of the DNA dodecamer d(CGCAAATTTGCG) and its complex with distamycin. Proc. Natl. Acad. Sci. U.S.A. 1987; 84:8385–8389. 3479798 10.1073/pnas.84.23.8385 PMC299547

[101] Nelson H.C.M., Finch J.T., Luisi B.F., Klug A. The structure of an oligo(dA)·oligo(dT) tract and its biological implications. Nature. 1987; 330:221–226. 3670410 10.1038/330221a0

