# Supplementary material for: Oxazinomycin arrests RNA polymerase at the polythymidine sequences
Source: Nucleic Acids Res. 2019 Sep 9;47(19):10296–312. doi: 10.1093/nar/gkz782 (PMC6821320; doi:10.1093/nar/gkz782)
Supplement: gkz782_Supplemental_File [file gkz782_supplemental_file.pdf]

Supplementary Table 1. DNA oligonucleotides and RNA primers used in this study

Name	type	Sequence (5'→3')	Employment in Figures
S274	tDNA	GCTCTCATCTGGACATGTGCACTCCTCTTAAACCTTAGATCGCTACAGTC	2,5
S275	ntDNA	GACTGTAGCGATCTAAGGTTTAAGAGGAGTGCACATGTCCAGATGAGAGC	2,5
S306M*	tDNA	GCTACTCTACGTCACTAT**X**CACTCCTCTTAAACCTTAGATCACTACAAGT	3,4
S307	ntNA	ACTTGTAGTGATCTAAGGTTTAAGAGGAGTGCATAGTGACGTAGAGTAGC	3,4
S244	tDNA	Atto680-TGGTGTCTGCTGTCTGTACGTCTCCTCTGGCCGTCTGTGCTCGTGTCTGG	6
S245	ntDNA	CCAGACACGAGCACAGACGGCCAGAGGAGACGTACAGACAGCAGACACCA	6
S332	tDNA	TGGTGATCTGGCTCTCATCTGGTCATGTGCGCTCCTCTTAAACCTTAGATCG	6,8,S6
S333	ntDNA	CGATCTAAGGTTTAAGAGGAGCGCACATGACCAGATGAGAGCCAGATCACCA	6,8,S6
S321	tDNA	GTACTGTTACTGATACTAGTGTACGCATGCGAGTCTAATCTGTTCTGCTCTCCTCTTAAACCTTACACTG	7
S322	ntDNA	CAGTGTAAGGTTTAAGAGGAGAGCAGAACAGATTAGACTCGCATGCGTACACTAGTATCAGTAACAGTAC	7
S323	tDNA	GTACTGTTACTGATACTAGTGTACGCATGCGAGTCAAATCTGTTCTGCTCTCCTCTTAAACCTTACACTG	7
S324	ntDNA	CAGTGTAAGGTTTAAGAGGAGAGCAGAACAGATTTGACTCGCATGCGTACACTAGTATCAGTAACAGTAC	7
S325	tDNA	GTACTGTTACTGATACTAGTGTACGCATGCGAGTAAAATCTGTTCTGCTCTCCTCTTAAACCTTACACTG	7,8
S326	ntDNA	CAGTGTAAGGTTTAAGAGGAGAGCAGAACAGATTTTACTCGCATGCGTACACTAGTATCAGTAACAGTAC	7,8
S345	tDNA	GTACTGTTACTGATACTAGTGTACGCATGCGAAAAAAATCTGTTCTGCTCTCCTCTTAAACCTTACACTG	7
S346	ntDNA	CAGTGTAAGGTTTAAGAGGAGAGCAGAACAGATTTTTTTCGCATGCGTACACTAGTATCAGTAACAGTAC	7
S272	tDNA	CGTCTCATCTGGCATCATCGCTCCTCTTAAACCTTAGATCGCTACAGTCG	S4
S273	ntDNA	CGACTGTAGCGATCTAAGGTTTAAGAGGAGCGATGATGCCAGATGAGACG	S4
S276	tDNA	GCCTTCATCTGCACAGCACGTCTCCTCTTAAACCTTAGATCGCTACAGTC	S4
S277	ntDNA	GACTGTAGCGATCTAAGGTTTAAGAGGAGACGTGCTGTGCAGATGAAGGC	S4
S001	tDNA	GTCTCATCTGGCATTGTACCTCCTCTTAAACCTTAGATCGCTACAGTC	S4
S028	ntDNA	GACTGTAGCGATCTAAGGTTTAAGAGGAGGTACAATGCCAGATGAGAC	S4
S312M*	tDNA	GCTACTCTACTGACATGTGCACTCCTCT**X**GAACCTTAGATCGCTACAAGT	S5
S313	ntDNA	ACTTGTAGCGATCTAAGGTTCCAGAGGAGTGCACATGTCAGTAGAGTAGC	S5
R002	RNA	Atto680-CACUAACUAAGAGGAG	2,3,4,5,6,7,8 S4,S6
R024	RNA	Atto680-CUCACAACCAGAGGAG	6,S5

***X** = 6-methyl-isoxanthopterin

Supplementary Table 2. *E. coli* protein expression vectors used in this study

Name	Description	Source/reference
pVS10	wild-type RNAP (T7p-α-β-β'_His$_6$-T7p-ω)	(Belogurov *et al*, 2007)
pGB163	Δ213 *Hsa* MT RNAP (T7p-His$_6$-Δ213 *Hsa* MT RNAP)	this work
pIA528+pIA839	β'N458S RNAP (T7p-α-β-β'_His$_6$) + *araB*p-ω	(Svetlov et al, 2004)
pIA578	GreA (T7p-GreA_His$_6$)	(Furman *et al*, 2013)
pIA577	GreB (T7p-GreB_His$_6$)	(Perederina *et al*, 2006)

Supplementary references

Belogurov G.A., Vassylyeva M.N., Svetlov V., Klyuyev S., Grishin N.V., Vassylyev D.G. & Artsimovitch I. (2007) Structural basis for converting a general transcription factor into an operon-specific virulence regulator. *Mol Cell.* 26(1):117-29

Furman R., Tsodikov O.V., Wolf Y.I. & Artsimovitch I (2013) An insertion in the catalytic trigger loop gates the secondary channel of RNA polymerase. *J. Mol. Biol.* 425: 82–93

Perederina A.A., Vassylyeva M.N., Berezin I.A., Svetlov V., Artsimovitch I. & Vassylyev D.G. (2006) Cloning, expression, purification, crystallization and initial crystallographic analysis of transcription elongation factors GreB from *Escherichia coli* and Gfh1 from *Thermus thermophilus*. *Acta Crystallogr. Sect. F Struct. Biol. Cryst. Commun.* 62: 44–46

Svetlov V., Vassylyev D.G. & Artsimovitch I. (2004) Discrimination against deoxyribonucleotide substrates by bacterial RNA polymerase. *J. Biol. Chem.* 279(37): 38087-90

A

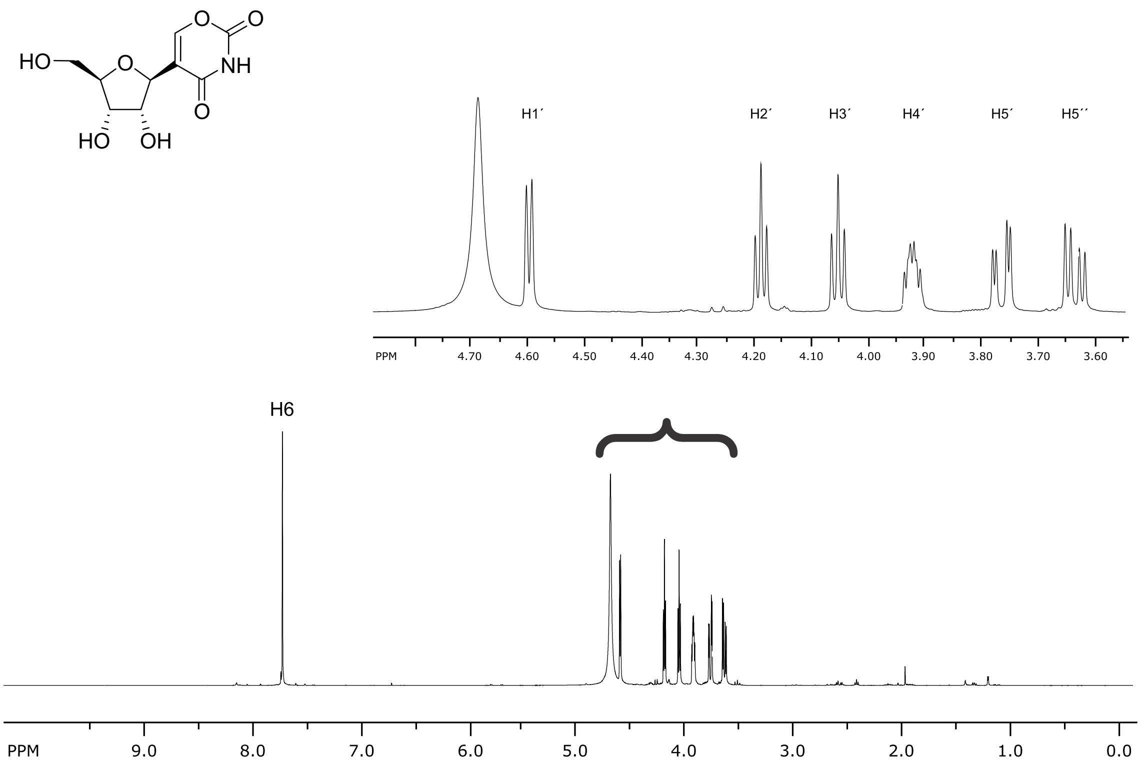

B

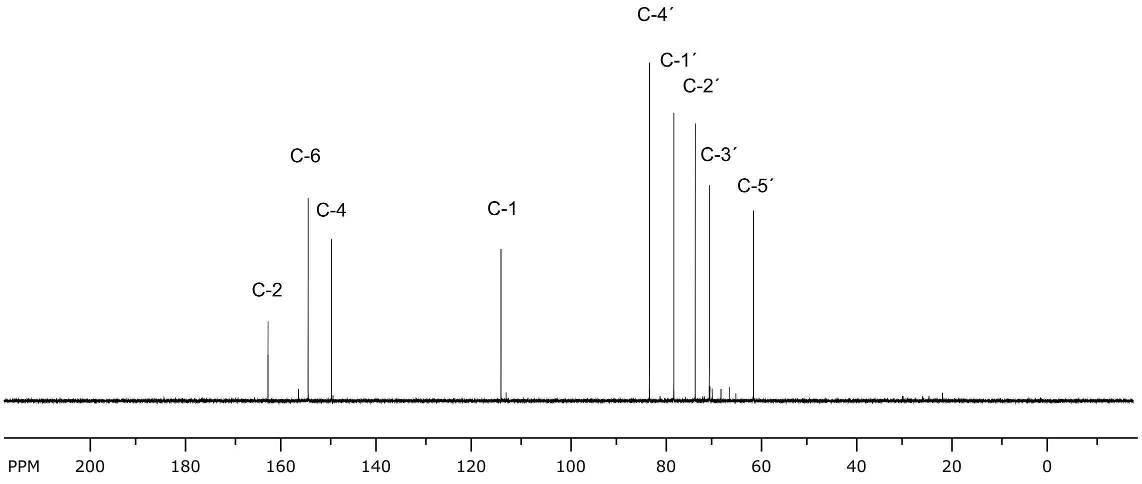

Supplementary Figure S1. Validation of the OZM chemical structure. The NMR spectra were recorded with a Bruker Avance 500 MHZ spectrometer. **(A)** 500 MHz (D_2O) ^1H NMR spectra of OZM. **(B)** 125 MHz (D_2O) ^{13}C NMR spectra of OZM.

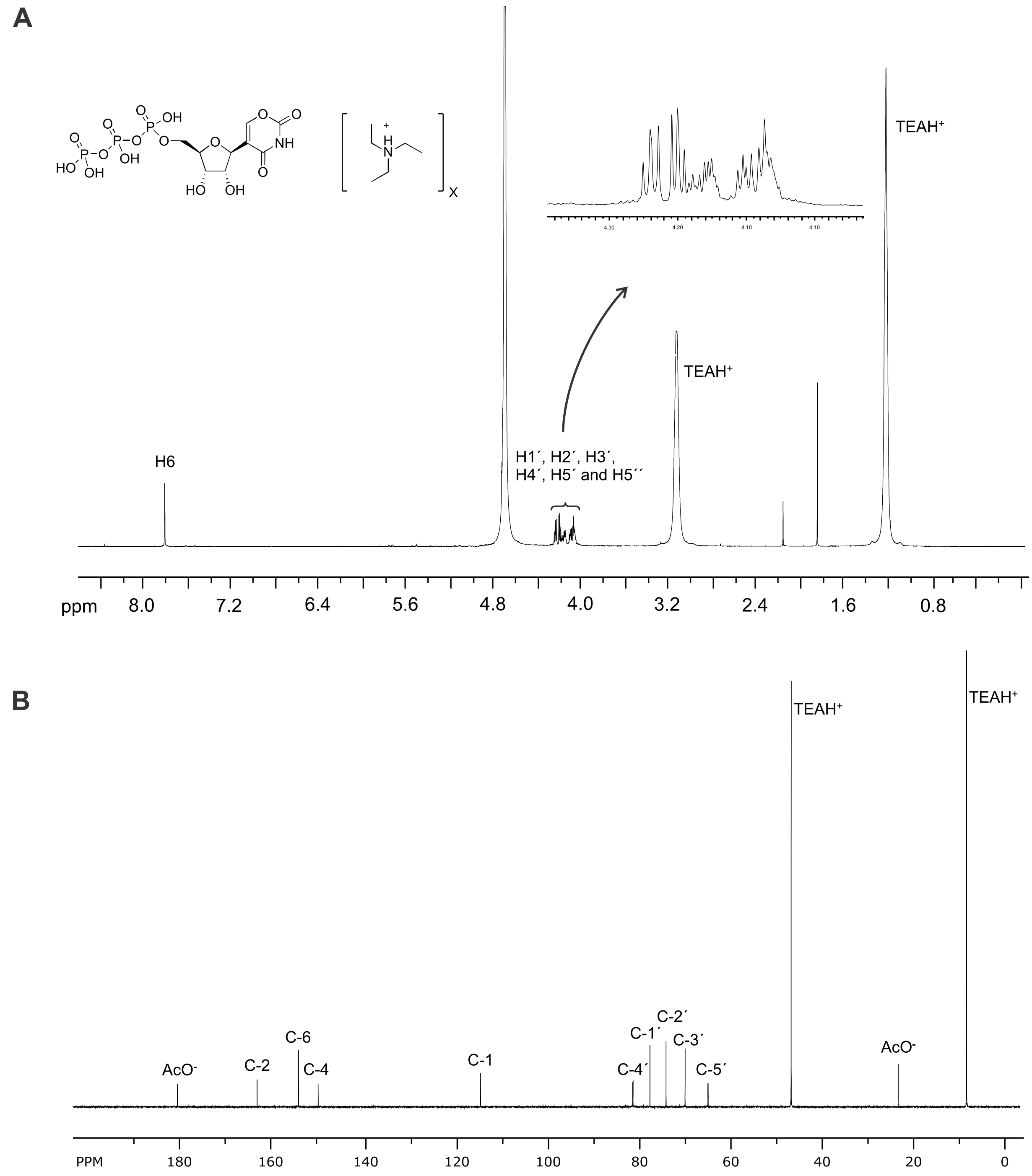

Supplementary Figure S2. Validation of the chemical structure of the OZM triphosphate. The NMR spectra were recorded with a Bruker Avance 500 MHZ spectrometer. **(A)** 500 MHz (D_2O) ^1H NMR spectra of the OZM triphosphate. **(B)** 125 MHz (D_2O) ^{13}C NMR spectra of the OZM triphosphate.

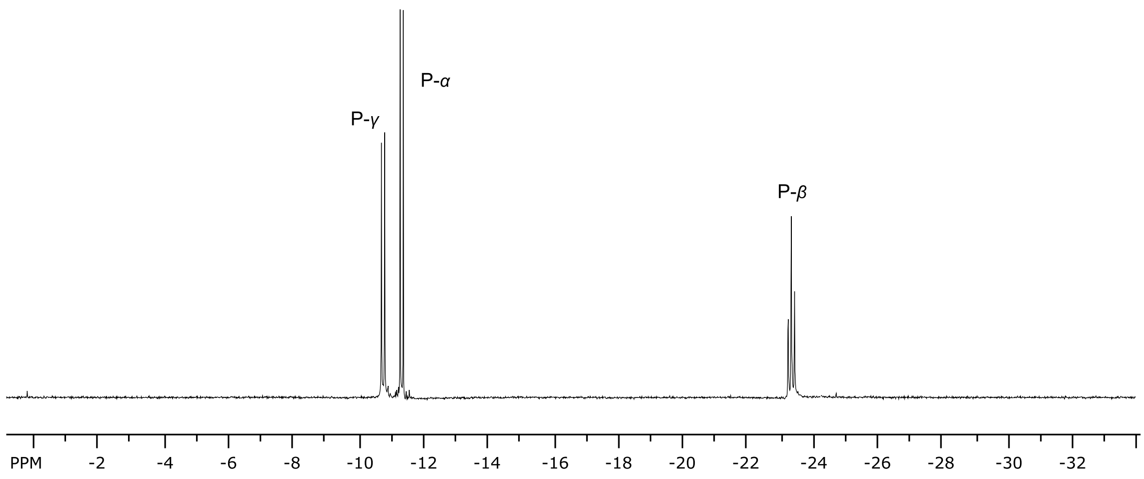

Supplementary Figure S3. 202 MHz (D_2O) ^{31}P NMR spectra of OZM 5´-triphosphate. The NMR spectra were recorded with a Bruker Avance 500 MHZ spectrometer.

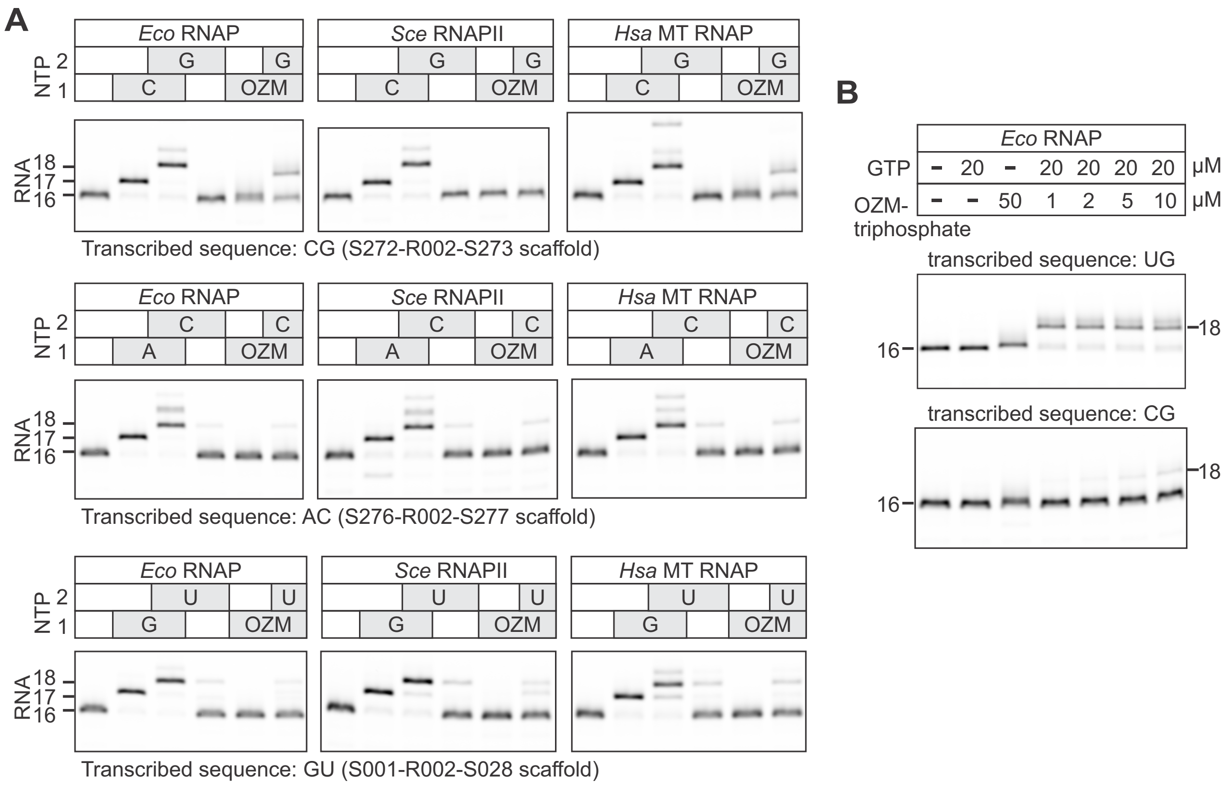

Supplementary Figure S4. *Eco* RNAP and *Hsa* MT RNAP inefficiently incorporate OZM in place of cytosine. NTPs were added at 20 µM unless indicated otherwise and the reactions were incubated for 2 min at 25°C. The identifiers of oligonucleotides used for TEC assembly are indicated for each experiment. The sequences of scaffolds used to assemble the TECs are presented in **Supplementary Table 1**. Pixel counts were linearly scaled to span the full 8-bit grayscale range within each gel panel. Fractional misicorporations (additional bands) are evident in several lanes. Each experiment was performed in triplicate. **(A)** Incorporation of OZM in place of cytosine (*top row*), adenine (*middle row*) and guanine (*bottom row*). **(B)** Incorporation of OZM in place of uridine (*top gel*) and cytosine (*bottom gel*) at low concentrations of OZM triphosphate.

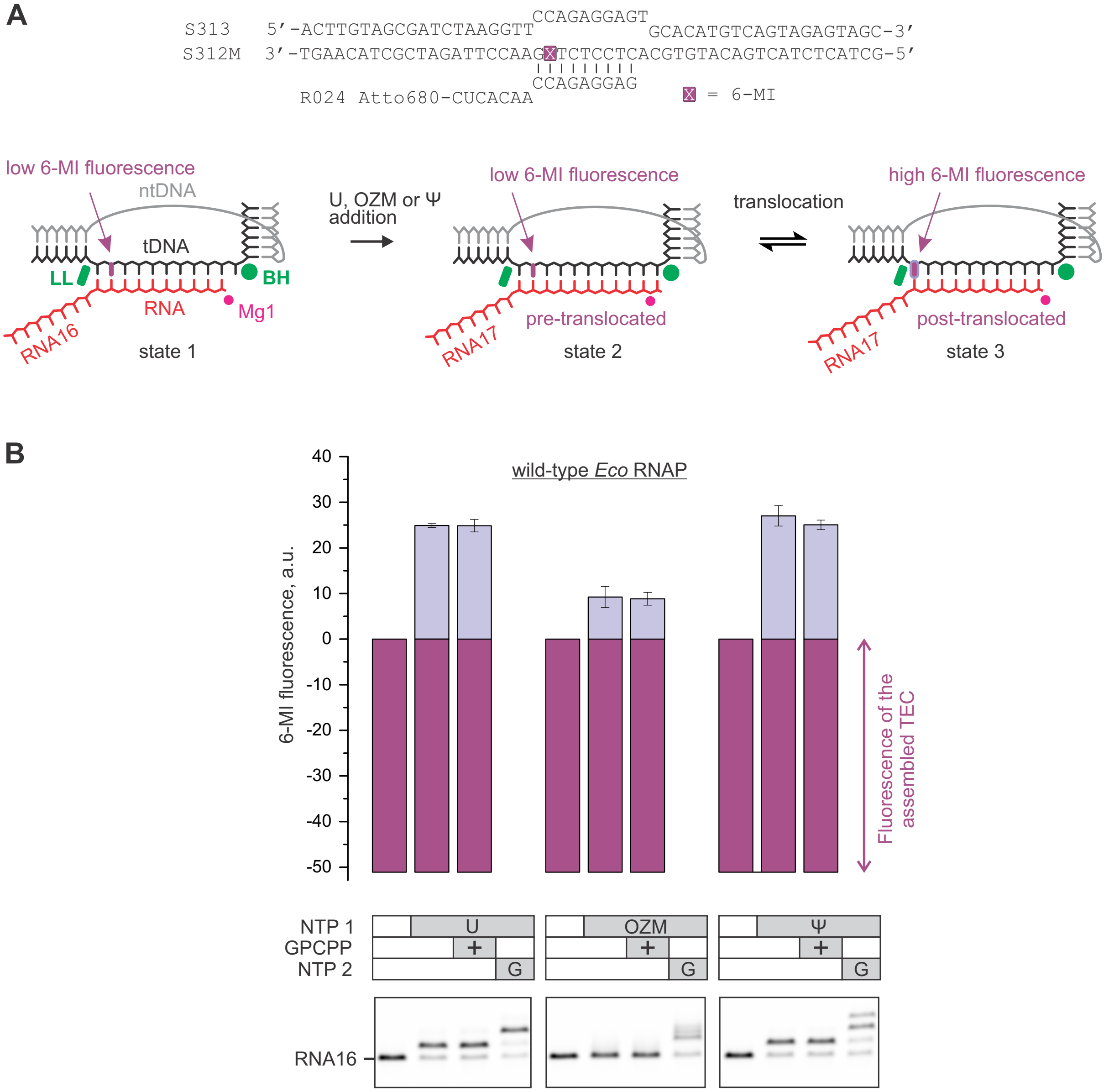

Supplementary Figure S5. The effect of OZM on translocation by *Eco* RNAP. (A) The nucleic acid scaffold employed in the translocation assay. The guanine analogue 6MI was initially positioned in the RNA:DNA hybrid eight nucleotides upstream of the RNA 3' end. The 6-MI fluorescence was quenched by the neighboring base pairs in the initial TEC (state 1) and the pre-translocated TEC that formed following the nucleotide incorporation (state 2) but increases when the 6-MI relocates to the edge of the RNA:DNA hybrid upon translocation (state 3). The Bridge Helix (BH) and the Lid loop (LL) are two structural elements of β' subunit that flank the RNA:DNA hybrid in the multisubunit RNAPs. **(B)** The fluorescence intensities observed upon incorporation of the uridine or OZM or Ψ by TECs assembled with the wild-type *Eco* RNAP. Gel panels reveal the length of the RNA at each step. The incorporation of OZM resulted in a very small change in the mobility of the RNA but was verified by further extension of the TEC with the guanosine.

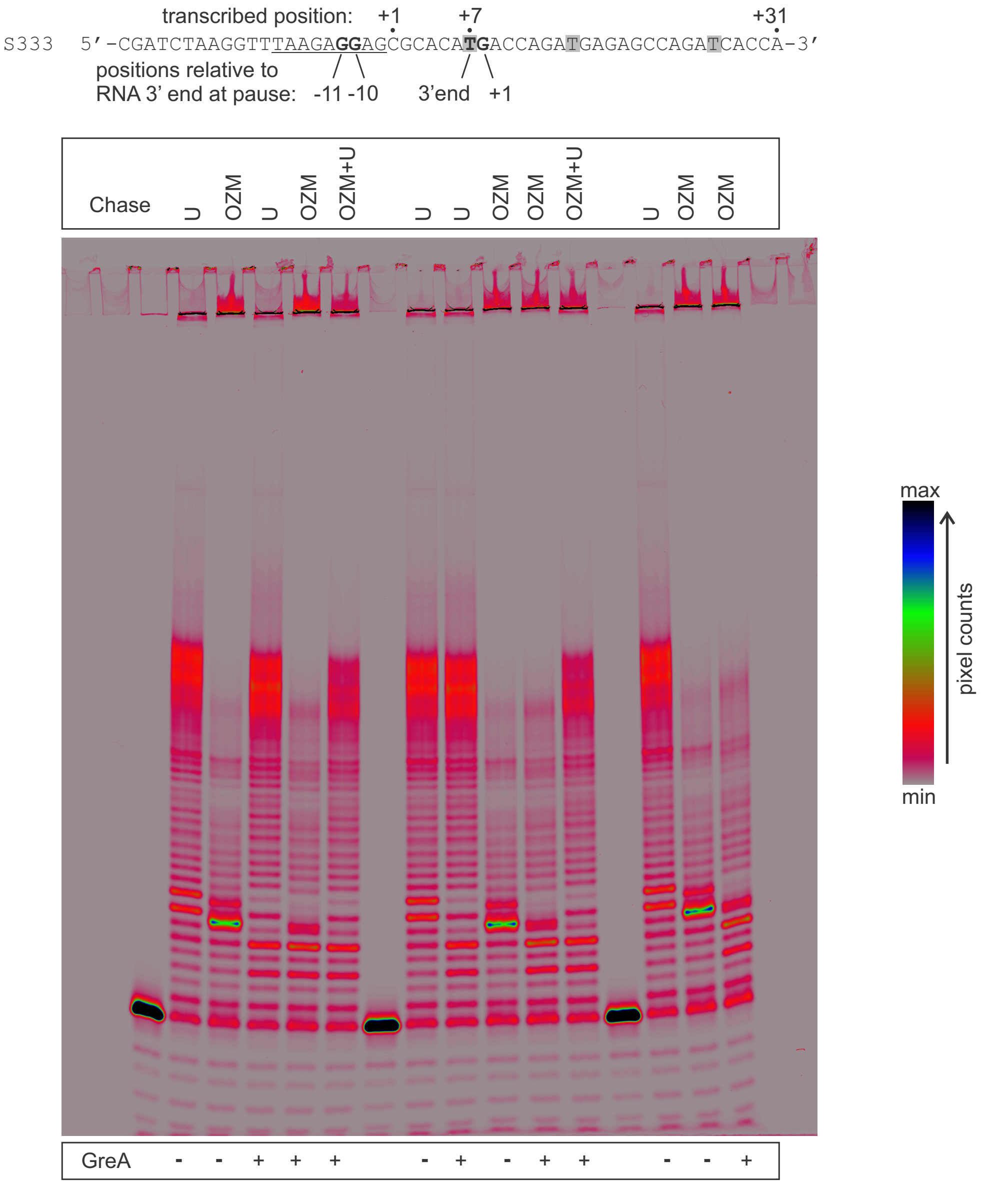

Supplementary Figure S6. Long OZM containing RNAs enter denaturing PAGE gels poorly. TECs were assembled using the scaffold shown above the gel (only the non-template DNA strand is shown, see Figure 5 for additional details) and chased with 100 μM ATP, CTP, GTP and UTP (U-chase) or OZM triphosphate (OZM-chase) or UTP and OZM triphosphate (OZM+U-chase) for 5 min at 25°C in the presence or absence of 2 μM GreA. The sequence corresponding to the annealing region of the RNA primer is underlined, thymidines in the transcribed region are highlighted. Lanes containing OZM-chase samples displayed larger amount of RNA in the gel wells than the U-chase lanes. Additional amount of the OZM containing RNA has likely diffused out of the wells into the buffer chamber. Pixel counts were linearly scaled to span 256 gradations within the gel image excluding the overexposed unchased samples. The gel was pseudocolored using RGB lookup table shown to the right of the gel.
